# On a Discrete SEIR Epidemic Model with Two-Doses Delayed Feedback Vaccination Control on the Susceptible

**DOI:** 10.3390/vaccines9040398

**Published:** 2021-04-18

**Authors:** Manuel De la Sen, Santiago Alonso-Quesada, Asier Ibeas, Raul Nistal

**Affiliations:** 1 Faculty of Science and Technology, Institute of Research and Development of Processes IIDP, University of the Basque Country, Barrio Sarriena, 48940 Leioa, Spain; santiago.alonso@ehu.eus (S.A.-Q.); raul.nistal@gmail.com (R.N.); 2 Department of Telecommunications and Systems Engineering, Universitat Autònoma de Barcelona, UAB, 08193 Barcelona, Spain; Asier.Ibeas@uab.cat

**Keywords:** discrete epidemic model, delayed feedback vaccination control, vaccination doses, COVID-19 pandemic

## Abstract

A new discrete susceptible-exposed-infectious-recovered (SEIR) epidemic model is presented subject to a feedback vaccination effort involving two doses. Both vaccination doses, which are subject to a non-necessarily identical effectiveness, are administrated by respecting a certain mutual delay interval, and their immunity effect is registered after a certain delay since the second dose. The delays and the efficacies of the doses are parameters, which can be fixed in the model for each concrete experimentation. The disease-free equilibrium point is characterized as well as its stability properties, while it is seen that no endemic equilibrium point exists. The exposed subpopulation is supposed to be infective eventually, under a distinct transmission rate of that of the infectious subpopulation. Some simulation examples are presented by using disease parameterizations of the COVID-19 pandemic under vaccination efforts requiring two doses.

## 1. Introduction

Typical formulations used to describe epidemic models are based on either differential or difference equations. In that way, the basic reproduction number and its physical and biological insight are discussed in [1] which is related to pertussis and measles descriptions. In addition, feedback vaccination laws have been developed using techniques such as sliding-mode control or linear or impulsive feedback vaccination [2,3]. The transient evolution of epidemic diseases is also an important issue for properly describing the day-to-day time-transmission levels and the appropriate eventual interventions to perform since the stability properties are more related to the stationary states, typically the disease-free and the endemic ones. For instance, in [4], an analytic methodology is given to predict and monitor the dates of maximum hospital occupancy of beds. The differential and difference models have also been corrected with other powerful analysis techniques. In that way, the bifurcation analysis and the stability of a fractional order susceptible-infectious-recovered (SIR) epidemic model with delay has been discussed in [5]. On the other hand, discretized and discrete-time epidemic models have been proposed in the background literature. The different approaches can basically consist of the discretization of continuous-time-based models by numerical methods or in the development of discrete models based in difference equations. See, for instance, [6,7,8,9,10,11,12] and some of the references therein. It turns out that since the relevant time of the dynamics evolution in epidemics is relatively long, typically for instance, on the orders of days or weeks, discrete-oriented epidemic models can be found to be appropriate for describing and monitoring the infection evolution through time. An extended SEIR model which incorporates its usual subpopulations, the asymptomatic, and the dead-infective subpopulations has been proposed in [6]. That model has been proposed as appropriate for the Ebola disease. On the other hand, a multistaged SIR was discussed in [7]. Such a model considers several coupled layers of infectious subpopulation for a coupled disease transmission from each layer to the adjacent ones. Moreover, discrete susceptible-infectious-recovered-susceptible (SIRS) models have also been studied in heterogeneous networks [8]. The vaccination effort can be considered either as an external forcing term or as a generator of a new subpopulation, the so-called vaccinated one. Such a subpopulation becomes dynamically coupled to the remaining ones in the model rather than as a specific forcing control [9]. Other types of epidemic models, such as, for instance, discretized susceptible-infectious-recovered (SIR)-type ones, or susceptible-infectious-susceptible (SIS)-type ones, have been proposed in [10,11,12,13] and some of the references therein.

Recently, a lot of research is being dedicated to studying and monitoring the new COVID-19 pandemic using registered data, like infection and detection tests, hospital bed occupancies, and mortality-related records. Normally, such data are updated in discrete time, typically, day to day or week to week. Therefore, discrete epidemic models have been found appropriate for processing such data. See, for instance, [14,15,16,17,18,19,20,21,22,23,24,25,26,27,28,29,30,31,32,33,34,35,36] and some related references therein. Studies on particular data for different countries or regions can be found in the literature related to COVID-19, sometimes related to public interventions, such as quarantines, isolation measures, lockdowns, use of masks, social distance rules, etc. See, for instance, those concerned with Saudi Arabia [16,17], Madrid capital town, metropolitan area and surrounding administrative area [18,19], India [23,24], Italy [25], United States [26], Canada and several of its provinces [28], Switzerland, [29], Brazil [30], etc. In addition, the analysis of data has been sometimes accompanied with mathematical analysis techniques on the pandemic evolution related to public interventions or mathematically founded analysis of the obtained data. In that way, the impact of lockdowns is investigated in [17], while the effects of total or partial quarantines are investigated in [18] for a SEIAR model, which incorporates the asymptomatic subpopulation to the typical SEIR model by considering the isolated population as removed either from the infectious individuals or from the susceptible ones. In [19], a more general model of potential usefulness in the description and monitoring of COVID-19 has been proposed, discussed, and tested with recorded real data. Such a model includes three different infectious subpopulations, namely, the slightly infections, the hospitalized ones and the ones staying at the intensive care units are considered. On the other hand, the implementation of control rules oriented either toward reducing the number of exposed individuals or toward increasing the number of treated individuals is proposed and discussed in [20] while impulsive optimal control techniques are developed in [21]. In particular, the analysis technique proposed in [20] relies on the fact that the epidemics is endemic. In [23], the inadequacy of the implementation of open-loop (i.e., without using feedback) controls is emphasized contrarily to the use of closed-loop controls like, for instance, sliding mode-based control or other feedback laws. Moreover, it has been proved in [27] that the suppression strategies are appropriate, provided that they are sufficiently strong while taken through prompt decisions, whereas the mitigation strategies can fail because of eventual unfavorable combinations of delays, unstable dynamics, and uncertainties in the model.

This paper proposes and investigates a new discrete SEIR model subject to a linear two-stage delayed feedback vaccination effort having in mind that some of the recently approved vaccines for COVID-19 require two doses for increasing their average effectiveness. Such doses are administered to the susceptible subpopulation with a delay period, and their potential benefits on immunity appear several days after the administration of the second doses. In fact, the model considers the injection of two doses with different delays and eventual average different effectiveness. The proposed model considers also that the exposed have a transmission rate exposed-susceptible which may be eventually distinct from the infectious-susceptible transmission rate. The underlying idea is that in some infectious diseases, such as, for instance, in the COVID-19 pandemic, there are contagions from both exposed to susceptible and from infectious to susceptible. The delays of the vaccination and expected achievable immunity as well as the transmission rates are model parameters which can be updated for different experiments, [37,38,39].

The paper is organized as follows: Section 2 is devoted to present the new mentioned proposed discrete susceptible-exposed-infectious-recovered (SEIR) epidemic model with delayed double-dose linear feedback vaccination. Section 3 discusses the non-negativity and boundedness of the solution under any given finite non-negative initial conditions, as well as the existence and components of the disease-free equilibrium point and its stability properties. It is also proved that the endemic equilibrium point does not exist for the proposed model in Section 4. Section 5 presents and discusses some examples of the proposed model related to the evolution of the COVID-19 pandemic. Finally, a set of illustrative concluding remarks ends the paper. Some auxiliary technical results are proved in Appendix A which are supported by general necessary mathematical results given or proved in [40,41,42,43,44].

## 2. The Discrete SEIR Epidemic Model Subject to Two Vaccination Doses

Note that the rationale of the sampling period interpretation is unity, typically one day or one week for a correct practical use of the model. The model parameters should be expressed in values of dimensionality being the inverse of the sampling period units. The SEIR epidemic model equations may be rewritten equivalently as follows:

(1) $$S_{k+1}=\left(a_{k}-\beta_{k}I_{k}-\beta_{k}^{e}E_{k}\right)S_{k}-V_{2,k-d_{2}}-V_{1,k-d_{1}-d_{2}}$$

(2) $$E_{k+1}=\left(1-\mu \right)E_{k}+\left(\beta_{k}I_{k}+\beta_{k}^{e}E_{k}\right)S_{k}$$

(3) $$I_{k+1}=\left(1-\gamma \right)I_{k}+\mu E_{k}$$

(4) $$R_{k+1}=R_{k}+\gamma I_{k}+V_{2,k-d_{2}}+V_{1,k-d_{1}-d_{2}}$$

(5) $$V_{2,k-d_{2}}=K_{k-d_{2}}\rho_{2}S_{k-d_{2}}$$

(6) $$V_{1,k-d_{1}-d_{2}}=K_{k-d_{1}-d_{2}}\rho_{1}S_{k-d_{1}-d_{2}}$$

for any integer $k\in Z_{0+}=Z_{+}\cup \left\{0\right\}$ and any given finite initial conditions $S_{0}\geq 0$, $E_{0}\geq 0$, $I_{0}\geq 0$ and $R_{0}\geq 0$ and $S_{k}=E_{k}=I_{k}=R_{k}=0$ for $k\in Z_{-}$, where S, E, I and R are the susceptible, exposed, infectious and recovered subpopulations, respectively. The forcing terms of Equations (5) and (6) are the two doses of vaccination on the susceptible which are generated via linear feedback and which are subject to integer delays $d_{1}+d_{2}$ (first dose) and $d_{2}$ (second dose), respectively, with respect to each current sampling instant, where $\min\left(d_{1},d_{2}\right)>0$. In the above model:

– $a_{k}$ is the average recruitment rate proportional to the susceptible at the kth sampling instant related; for instance, to the rates of births and *a* is a constant reference value for the above sequence; for instance, its average over the whole time period under study and typically it might be unity.
– $\beta_{k}$ and $\beta_{k}^{e}$ are, respectively, the average transmission rates of the infectious and exposed subpopulations at the k−th sampling instant.
– γ is the average recovery rate.
– μ is the average incubation rate.
– $K_{k}$ is the vaccination rate (a feedback control gain) which can be eventually depending on the sampling instants. It is assumed in the sequel that ${\left\{K_{k}\right\}}_{0}^{\infty }\subset \left[0\;,\;1\right]$.
– $\rho_{1}$, $\rho_{1}+\rho_{2}$ are parameters in (0 , 1] which quantify the average effectiveness (or efficiency) of the respective doses. In particular, $\rho_{2}$ gives the extra effectiveness obtained from the injection of the second dose. In this context, $\rho_{1}+\rho_{2}\in \left[0\;,\;1\right]$ and $\rho_{1}+\rho_{2}=1$ refer to the ideal situation, unattainable in practice, of 100 percent effectiveness of the combined injection of the two doses. Note that $\rho_{1}=\rho_{2}=\rho_{1}+\rho_{2}=0$ refers to the worst case where the vaccination is fully superfluous.

Typically, we can consider two different transmission rates for the exposed and infectious since the exposed are not usually identified to be allocated under quarantine or isolation, and furthermore, it has been argued that the infective periods are, in general, of distinct time length for both stages in the case of COVID-19 pandemic. It turns out that these transmission rates can be time varying, in general, since the transmission rates can depend on the intervention measures and on the social customs in the geographic area under study. Note that, even if the vaccination is performed under the same gain  K for both doses, it can be considered that the effectiveness if only the first injected dose is smaller than if both of them are injected. Therefore, it can be typically argued that $\rho_{2}>0$ leads to $\rho_{1}+\rho_{2}>\rho_{1}.$ It can also be pointed out that the proposed discrete-time model corresponds to the backward Euler discretization on the continuous –time SEIR model given by:

$$\begin{array}{l} \dot{S}\left(t\right)=\nu \left(t\right)S\left(t\right)-\left(\beta^{\prime }I\left(t\right)+\beta^{\prime }{}^{e}E\left(t\right)\right)S\left(t\right)-{V_{1}}^{\prime }\left(t-d_{1}-d_{2}\right)-{V_{2}}^{\prime }\left(t-d_{2}\right)\\ \dot{E}\left(t\right)=-\mu^{\prime }E\left(t\right)+\left(\beta^{\prime }I\left(t\right)+\beta^{\prime }{}^{e}E\left(t\right)\right)S\left(t\right)\\ \dot{I}\left(t\right)=\mu^{\prime }E\left(t\right)-\gamma^{\prime }I\left(t\right)\\ \dot{R}\left(t\right)=\gamma^{\prime }I\left(t\right)+{V_{1}}^{\prime }\left(t-d_{1}-d_{2}\right)+{V_{2}}^{\prime }\left(t-d_{2}\right) \end{array}$$

whose discretization is given by

$$\begin{array}{l} S_{k+1}=S_{k}+h\nu_{k}S_{k}-\left(h\beta^{\prime }I_{k}+h\beta^{\prime }{}^{e}E_{k}\right)S_{k}-h{V_{1,k-d_{1}-d_{2}}}^{\prime }-h{V_{2,k-d_{2}}}^{\prime }\\ =\left(1+h\nu \right)S_{k}-\left(h\beta^{\prime }I_{k}+h\beta^{\prime }{}^{e}E_{k}\right)S_{k}-h{V_{1,k-d_{1}-d_{2}}}^{\prime }-h{V_{2,k-d_{2}}}^{\prime }\\ E_{k+1}=E_{k}-h\mu^{\prime }E_{k}+\left(h\beta^{\prime }I_{k}+h\beta^{\prime }{}^{e}E_{k}\right)S_{k}=\left(1-h\mu^{\prime }\right)E_{k}+\left(h\beta^{\prime }I_{k}+h\beta^{\prime }{}^{e}E_{k}\right)S_{k}\\ I_{k+1}=I_{k}+h\mu^{\prime }E_{k}-h\gamma^{\prime }I_{k}=\left(1-h\gamma^{\prime }\right)I_{k}+h\mu^{\prime }E_{k}\\ R_{k+1}=R_{k}+h\gamma^{\prime }I\left(t\right)+h{V_{1,k-d_{1}-d_{2}}}^{\prime }+h{V_{2,k-d_{2}}}^{\prime } \end{array}$$

so that the discretized and the proposed discrete-time model are equivalent if the following correspondence is performed

$$\begin{array}{l} a_{k}=1+h\nu_{k}\\ \beta =h\beta^{\prime },\beta^{e}=h\beta^{\prime }{}^{e}\\ V_{1,k-d_{1}-d_{2}}=h{V_{1,k-d_{1}-d_{2}}}^{\prime },V_{2,k-d_{2}}=h{V_{2,k-d_{2}}}^{\prime }\\ \mu =h\mu^{\prime },\gamma =h\gamma^{\prime } \end{array}$$

However, the model is originally set up in discrete-time instead as the discretization of a continuous-time one. One of the daunting challenges to counteract the epidemic spreading is the design of the vaccination functions. The control theory provides a framework to systematically design them in order to fulfill with a prescribed control objective. Thus, an output feedback approach is adopted in this work and discussed in the sequel. However, control theory has developed many analytical tools that allows one to face this problem by using other approaches such as state feedback and feedback linearization [2], output-controllability [19], optimal control [21], impulsive control [22], sliding mode [23] and lockdown and quarantine [18,28], to cite just a few.

The model (1)–(6) may be compacted after replacing the vaccination controls (5)–(6) in the susceptible and recovered subpopulations dynamics, which result in:

(7) $$S_{k+1}=aS_{k}-K\left(\rho_{2}S_{k-d_{2}}+\rho_{1}S_{k-d_{1}-d_{2}}\right)+u_{k}$$

(8) $$E_{k+1}=\left(1-\mu \right)E_{k}+\left(\beta_{k}I_{k}+\beta_{k}^{e}E_{k}\right)S_{k}$$

(9) $$I_{k+1}=\left(1-\gamma \right)I_{k}+\mu E_{k}$$

(10) $$R_{k+1}=R_{k}+\gamma I_{k}+K\left(\rho_{2}S_{k-d_{2}}+\rho_{1}S_{k-d_{1}-d_{2}}\right)-g_{k}$$

(11) $$u_{k}=\left[a_{k}-a-\beta_{k}\left(I_{k}+\lambda_{k}^{e}E_{k}\right)\right]S_{k}+g_{k}$$

(12) $$g_{k}=\left(K-K_{k-d_{2}}\right)\rho_{2}S_{k-d_{2}}+\left(K-K_{k-d_{1}-d_{2}}\rho_{1}\right)S_{k-d_{1}-d_{2}}$$

for any integer $k\in Z_{0+}$, where $\lambda_{k}^{e}=\beta_{k}^{e}/\beta_{k}$ and K and a are reference values for the vaccination rate gain and recruitment, for instance, the estimated average values of their asymptotic limits if they converge. In particular, a scalar discrete function of the form of the susceptible subpopulation (7) is discussed in detail in Appendix A from the points of view of stability and convergence to a limit. Those auxiliary results based on a similar discrete Equation (7) subject to (11) are then used to discuss the stability of the proposed epidemic model.

## 3. Non-Negativity, Stability and Disease-Free Equilibrium Point

The following result which relies on boundedness, convergence and non-negativity of the solution is direct by summing up (7) to (10) (or (1) to (4)) by considering (7) to (11) (or (1) to (6)):

**Theorem** **1**. *The following properties hold:*

(i) *Assume that*$N_{0}=S_{0}+E_{0}+I_{0}+R_{0}=1$. *Then, the total population*$\;N_{k}=S_{k}+E_{k}+I_{k}+R_{k}=1$; $\forall k\in Z_{0+}$*if*$a_{k}\equiv 1$.
  $$(\mathrm{ii})\;N_{k}=1+\sum_{j=0}^{k-1}\left(a_{j}-1\right)S_{j};\;\forall k\in Z_{0+}$$
(iii) ${\left\{S_{k}\right\}}_{k=0}^{\infty }$*is non-increasing, and then bounded and convergent, if*
  (13) $$V_{2,k-d_{2}}+V_{1,k-d_{1}-d_{2}}=K_{k-d_{2}}\rho_{2}S_{k-d_{2}}+K_{k-d_{1}-d_{2}}\rho_{1}S_{k-d_{1}-d_{2}}\geq \left(a_{k}-\beta_{k}\left(I_{k}+\lambda_{k}^{e}E_{k}\right)-1\right)S_{k};\;\forall k\in Z_{0+}$$
  *leading to*
  (14) $$1+S_{k-1}\sum_{j=0}^{k-1}\left(a_{j}-1\right)\leq N_{k}\leq 1+S_{0}\sum_{j=0}^{k-1}\left(a_{j}-1\right);\forall k\in Z_{0+}$$
  *and, if the average value of the sequence*${\left\{a_{k}\right\}}_{k=0}^{\infty }\;$*is unity, then there exists the limit*$\underset{k\to \infty }{lim}N_{k}=N_{0}=1$. ${\left\{S_{k}\right\}}_{k=0}^{\infty }$*is strictly decreasing if the inequality in (13) is strict.*
(iv) ${\left\{S_{k}\right\}}_{k=0}^{\infty }$*is non-negative if and only if*
  (15) $$V_{2,k-d_{2}}+V_{1,k-d_{1}-d_{2}}=K_{k-d_{2}}\rho_{2}S_{k-d_{2}}+K_{k-d_{1}-d_{2}}\rho_{1}S_{k-d_{1}-d_{2}}\leq \left(a_{k}-\beta_{k}\left(I_{k}+\lambda_{k}^{e}E_{k}\right)\right)S_{k}\;;\;\forall k\in Z_{0+}$$
  *provided that the infectious transmission rate is sufficiently small according to*$\beta_{k}\leq \frac{a_{k}}{I_{k}+\lambda_{k}^{e}E_{k}{}^{}}$; $\forall k\in Z_{0+}$*which is guaranteed if*$\beta_{k}\leq \frac{a_{k}}{{\left(1+\lambda_{k}^{e}\right)}^{}N_{k}}$; $\forall k\in Z_{0+}$.
(v) *Assume that*μ∈[0 , 1), γ∈[0 , 1)*and that (15) holds. Then, any sequence trajectory solution of (1)–(4) subject to (5)–(6) is non-negative.*

**Proof.**  If $a_{k}=1$; $\forall k\in Z_{0+}$ then $N_{0}=S_{0}+E_{0}+I_{0}+R_{0}=1$ implies directly that $N_{k}=S_{k}+E_{k}+I_{k}+R_{k}=N_{0}=1$; $\forall k\in Z_{0+}$ by summing up (1) to (4). The same calculations prove Property (ii) in the general case. To prove, Property (iii), note from (3) that, under the given constraint, ${\left\{S_{k}\right\}}_{k=0}^{\infty }$ is non-increasing so that (14) holds directly from Property (ii) since:

(16) $$\frac{S_{k+1}}{S_{k}}=a_{k}-\beta_{k}\left(I_{k}+\lambda_{k}^{e}E_{k}\right)-\frac{V_{2,k-d_{2}}+V_{1,k-d_{1}-d_{2}}}{S_{k}}\leq 1;\;\forall k\in Z_{0+}$$

Note that ${\left\{S_{k}\right\}}_{k=0}^{\infty }$ is also bounded and convergent since it is non-increasing and its initial condition is finite by hypothesis. Property (iv) follows directly by replacing (15) with (1) subject to $S_{0}\geq 0$ and to $\beta_{k}\leq \frac{a_{k}}{I_{k}+\lambda_{k}^{e}E_{k}{}^{}}$ (guaranteed if $\beta_{k}\leq \frac{a_{k}}{{\left(1+\lambda_{k}^{e}\right)}^{}N_{k}}$); $\forall k\in Z_{0+}$ since $V_{k}\geq 0$; $\forall k\in Z_{0+}$. If, addition, μ∈[0 , 1), γ∈[0 , 1) then from (2)–(4) and ${\left\{S_{k}\right\}}_{k=0}^{\infty }\subset R_{0+}=R_{+}\cup \left\{0\right\}$ yields ${\left\{E_{k}\right\}}_{k=0}^{\infty }\subset R_{0+}$, ${\left\{I_{k}\right\}}_{k=0}^{\infty }\subset R_{0+}$ and ${\left\{R_{k}\right\}}_{k=0}^{\infty }\subset R_{0+}$, since the initial conditions of all the subpopulations are non-negative. Property (v) is proved. □

**Remark** **1.** *The non-negativity property of the susceptible of Theorem 1 (iv) is a necessary constraint for a well-posedness of the model. Note that the condition of sufficiently smaller transmission rate of the Infectious* $\beta_{k}\leq \frac{a_{k}}{{\left(1+\lambda_{k}^{e}\right)}^{}N_{k}}$ *;* $\forall k\in Z_{0+}$ *, which guarantees according to Theorem 1 (iv) that the sequence of susceptible is non-negative if* $\;S_{0}\geq 0$ *and the vaccination satisfies (15), is reasonable since the transmission rate decreases as the total population increases in the popular class of true-mass action epidemic models. The property of the susceptible sequence being non-increasing [Theorem 1 (iii)] describes appropriately the disease growing since it starts until its first maximum peak.*

**Remark** **2.** *It turns out the need of the assumption*${\left\{K_{k}\right\}}_{0}^{\infty }\subset \left[0\;,\;1\right]$*for the vaccination gain sequence since the daily vaccination (or, in general, the one for the used sampling period in the model parameterization) is proportional to the susceptible subpopulation. Therefore, note that the condition (13) for*${\left\{S_{k}\right\}}_{k=0}^{\infty }$*to be non-increasing requires the necessary condition:*

(17) $$\rho_{2}S_{k-d_{2}}+\rho_{1}S_{k-d_{1}-d_{2}}\geq \left(a_{k}-\beta_{k}\left(I_{k}+\lambda_{k}^{e}E_{k}\right)-1\right)S_{k};\;\forall k\in Z_{0+}$$

*to be fulfilled by the vaccination gain unity, which is the maximum possible one, provided that the existing stock vaccines covers such a need. Note that the above condition always holds if*${\left\{S_{k}\right\}}_{k=0}^{\infty }$*is non-negative and*$a_{k}\leq \beta_{k}\left(I_{k}+\lambda_{k}^{e}E_{k}\right)+1$*;*$\forall k\in Z_{0+}$*and it also holds if*${\left\{S_{k}\right\}}_{k=0}^{\infty }$*is non-negative,*$a_{k}>\beta_{k}\left(I_{k}+\lambda_{k}^{e}E_{k}\right)+1$*;*$\forall k\in Z_{0+}$. *This second condition is guaranteed by under unity vaccination gain:*

(18) $$\frac{\left(\rho_{1}+\rho_{2}\right)\min\left(S_{k-d_{2}}\;,\;S_{k-d_{1}-d_{2}}\right)}{\left(a_{k}-\beta_{k}\left(I_{k}+\lambda_{k}^{e}E_{k}\right)-1\right)S_{k}}\geq 1;\forall k\in Z_{0+}$$

*In practice,*$\frac{\min\left(S_{k-d_{2}}\;,\;S_{k-d_{1}-d_{2}}\right)}{S_{k}}$*is slightly greater than unity if the susceptible sequence is decreasing since*$d_{1}$*and*$d_{1}+d_{2}$*are typically delays of one and three weeks, in the case of SARS-CoV-2 which do not affected significantly the variation of the susceptible population levels. In addition,*$a_{k}-\beta_{k}\left(I_{k}+\lambda_{k}^{e}E_{k}\right)-1$*is typically close to zero for small infection levels compared to the susceptible population values. Therefore, for reasonable vaccine efficacies, (18) holds if*$a_{k}>\beta_{k}\left(I_{k}+\lambda_{k}^{e}E_{k}\right)+1$*;*$\forall k\in Z_{0+}$.

*Note that the condition (15) for the susceptible sequence* ${\left\{S_{k}\right\}}_{k=0}^{\infty }$ *to be non-negative requires the necessary condition to be fulfilled by some maximum possible vaccination gain rate* K≤1 *(since this feature would ensure that it holds for any lower effort as well), that is,*

$\left(a_{k}-\beta_{k}\left(I_{k}+\lambda_{k}^{e}E_{k}\right)\right)S_{k}\geq 0$*;*$\forall k\in Z_{0+}$*which holds if*$S_{0}\geq 0$*if*$\beta_{k}\leq \frac{a_{k}}{I_{k}+\lambda_{k}^{e}E_{k}{}^{}}$*;*$\forall k\in Z_{0+}$*which is guaranteed if*$\beta_{k}\leq \frac{a_{k}}{{\left(1+\lambda_{k}^{e}\right)}^{}N_{k}}$*;*$\forall k\in Z_{0+}$.

The existence of a disease- free equilibrium point is discussed in the subsequent result:

**Theorem 2**. *Assume that*$\rho_{1}+\rho_{2}\leq 1$*,*$\beta_{k}\leq \frac{a_{k}}{I_{k}+\lambda_{k}^{e}E_{k}{}^{}}$*(guaranteed if*$\beta_{k}\leq \frac{a_{k}}{{\left(1+\lambda_{k}^{e}\right)}^{}N_{k}}$*);*$\forall k\in Z_{0+}$*,*${\left\{a_{k}\right\}}_{k=0}^{\infty }\to a\left(\leq 1\right)$*,*${\left\{K_{k}\right\}}_{k=0}^{\infty }\left(\subset \left[0\;,\;1\right]\right)\to K$*and assume also that the constraints (13) and (15) hold. Then, there is a disease-free equilibrium point*$x_{df}={\left(S_{df},\;0,0,R_{df}=N_{df}-S_{df}\right)}^{T}$*, being in general dependent on the initial conditions, with*$S_{df}\geq 0$*and*$R_{df}=N_{df}-S_{df}$*, where*${\left\{N_{k}\right\}}_{k=0}^{\infty }\to N_{df}$*, such that*$S_{df}=0$*if*$K\neq \frac{a}{\rho_{1}+\rho_{2}}$.

**Proof.**  Note that ${\left\{K_{k}\right\}}_{k=0}^{\infty }\left(\subset \left[0,\;1\right]\right)\to K$ implies that K∈[0, 1]. Note also that $E_{df}=0$ and $I_{df}=0$ are trivial solutions of (2) and (3) if ${\left\{S_{k}\right\}}_{k=0}^{\infty }$ is bounded. It follows furthermore that ${\left\{S_{k}\right\}}_{k=0}^{\infty }$ is non-increasing and bounded from Theorem 1 (iii), ${\left\{a_{k}\right\}}_{k=0}^{\infty }\to a\left(\leq 1\right)$ and (13) holds. As a result, if furthermore, (15) holds then ${\left\{S_{k}\right\}}_{k=0}^{\infty }\left(\subset R_{0+}\right)\to S_{df}\left(\geq 0\right)$ with $S_{df}=0$ if $K\neq \frac{a}{\rho_{1}+\rho_{2}}$ subject to the assumed constraint $\rho_{1}+\rho_{2}\geq 1$ since K∈[0, 1]. Note that

(19) $$N_{k+1}=a_{k}S_{k}+E_{k}+I_{k}+R_{k}=N_{k}+\left(a_{k}-1\right)S_{k}$$

implies that $N_{k+1}-N_{k}=\left(a_{k}-1\right)S_{k}$ and ${\left\{a_{k}\right\}}_{k=0}^{\infty }\to 1$ implies that ${\left\{N_{k}\right\}}_{k=0}^{\infty }\to N_{df}$ and ${\left\{R_{k}\right\}}_{k=0}^{\infty }\to R_{df}=N_{df}-S_{df}$ since ${\left\{E_{k}\right\}}_{k=0}^{\infty }\to E_{df}=0$ and ${\left\{I_{k}\right\}}_{k=0}^{\infty }\to I_{df}=0$. □

The subsequent result gives conditions for both the infection and the susceptibility to asymptotically vanish implying convergence of the solution to a disease-free equilibrium point without susceptible individuals. Some supportive auxiliary results used in its proof are given in Appendix A.

**Theorem** **3.** *Assume that the constraint (15) holds, so that any solution sequence is non-negative for non-negative initial conditions. Assume also that the first assumptions below, and, furthermore, at least one of the assumptions 2 to 5 below also holds:*

(1) μ∈[0 , 1)*,*γ∈[0 , 1)*,*$\rho_{1}+\rho_{2}\leq 1$*,*$K_{k}\leq \frac{a_{k}}{I_{k}+\lambda_{k}^{e}E_{k}}$*(guaranteed if*$\beta_{k}\leq \frac{a_{k}}{{\left(1+\lambda_{k}^{e}\right)}^{}N_{k}}$*);*$\forall k\in Z_{0+}$*,*${\left\{a_{k}\right\}}_{k=0}^{\infty }\to a\left(\leq 1\right)$*,*${\left\{\beta_{k}\right\}}_{k=0}^{\infty }\to \beta$*,*${\left\{\lambda_{k}^{e}\right\}}_{k=0}^{\infty }\to \lambda^{e}$*,*${\left\{K_{k}\right\}}_{k=0}^{\infty }\to K$.
(2) *Either the constraint (13) holds with strict inequality for all*$k\in Z_{0+}$*and*$K\neq \frac{a}{\rho_{1}+\rho_{2}}$.
(3) $K\in \left(0\;,\;\min\left(\;\overset{—}{K}\;,\;1\right)\;\right)$ *, where:*

(20) $$\overset{—}{K}=\sup\left\{y\in \left(\frac{a-1}{\rho_{1}+\rho_{2}}\;,\;\frac{1+a}{\rho_{1}+\rho_{2}}\right)\;:y<1/f\left(y\right)\right\}$$

(21) $$f\left(y\right)=\underset{\theta \in \left(0,2\pi \right)}{sup}\sqrt{\frac{{\left(\rho_{2}\left(\cos\left(\theta d_{2}\right)-1\right)+\rho_{1}\left(\cos\left(\theta \left(d_{1}+d_{2}\right)\right)-1\right)\right)}^{2}+{\left(\rho_{2}\sin\left(\theta d_{2}\right)+\rho_{1}\sin\left(\theta \left(d_{1}+d_{2}\right)\right)\right)}^{2}}{1+{\left(a-\left(\rho_{1}+\rho_{2}\right)y\right)}^{2}-2\left(a-\left(\rho_{1}+\rho_{2}\right)y\right)\cos\theta }}$$

*and*

(22) $$\left|K_{k}-K\right|=o\left(\|{\hat{x}}_{k}\|{}^{-1}\right);\;\left|a_{k}-a\right|=o\left(\|{\hat{x}}_{k}\|{}^{-1}\right)$$

*where*${\hat{x}}_{k}={\left(x_{k},\;x_{k-1},\ldots ,x_{k-d_{2}},\ldots ,x_{k-d_{1}-d_{2}}\right)}^{T}$.

(23) $$(4)\;a\in \left(0\;,\;1\right),\;K\in \left[0\;,\;1/\underset{\theta \in \left[0,2\pi \right)}{sup}\left|\frac{\rho_{2}e^{-i\theta d_{2}}+\rho_{1}e^{-i\theta \left(d_{1}+d_{2}\right)}}{e^{i\theta }-a}\right|\right)$$

*and (22) holds.*

(24) $$(5)\;a\in \left(0\;,\;1\right),K\in \left[0\;,\;\frac{\left(c_{1}^{-d_{1}}-a\right)c_{1}}{\rho_{1}+\rho_{2}c_{2}}\right)$$

*Then, there exists a globally asymptotically stable disease-free equilibrium point*$x_{df}={\left(0,\;0,0,R_{df}=N_{df}\right)}^{T}$*and*${\left\{N_{k}\right\}}_{k=0}^{\infty }\to N_{df}$.

**Proof.**  The above assumptions 1 and 2 guarantee that ${\left\{S_{k}\right\}}_{k=0}^{\infty }\to 0$, and that ${\left\{S_{k}\right\}}_{k=0}^{\infty }$ is bounded, from Theorem 1 and Theorem 2 for any given non-negative finite initial conditions. Note also that, from (2) and (3), ${\left\{E_{k}\right\}}_{k=0}^{\infty }\to 0$ and ${\left\{I_{k}\right\}}_{k=0}^{\infty }\to 0$ since μ∈[0, 1) and γ∈[0, 1).

In addition, any of the assumptions 3 or 4, together with the properties ${\left\{E_{k}\right\}}_{k=0}^{\infty }\to 0$ and ${\left\{I_{k}\right\}}_{k=0}^{\infty }\to 0$ guarantees that ${\left\{S_{k}\right\}}_{k=0}^{\infty }\to 0$, and ${\left\{S_{k}\right\}}_{k=0}^{\infty }$ is bounded, for any given non-negative finite initial conditions from Theorem A1 [(i)–(ii)] and Corollary A1 of Appendix A. The same result also holds under the assumption 5 according to Theorem A3 (i) of Appendix A.

Thus, the disease-free equilibrium point $x_{df}={\left(0,\;0,0,R_{df}=N_{df}\right)}^{T}$ exists under the joint assumptions 1 and 2 and under the assumption 1 jointly with one of the assumptions 3 to 5. Now, note that

Since ${\left\{\beta_{k}S_{k}\right\}}_{k=0}^{\infty }\to \beta S_{df}=0$, Equations (2) and (3) can be compacted as follows:

(25) $$\begin{array}{ll} \left[\begin{array}{c} E_{k+1}\\ I_{k+1} \end{array}\right] & =\left[\begin{array}{cc} 1-\mu +\lambda_{k}^{e}\beta_{k}S_{k} & \beta_{k}S_{k}\\ \mu & 1-\gamma \end{array}\right]\left[\begin{array}{c} E_{k}\\ I_{k} \end{array}\right]\\ & =\left[\begin{array}{cc} 1-\mu +\lambda^{e}\beta S_{df} & \beta S_{df}\\ \mu & 1-\gamma \end{array}\right]\left[\begin{array}{c} E_{k}\\ I_{k} \end{array}\right]+\left[\begin{array}{cc} \beta \left(\lambda_{k}^{e}S_{k}-\lambda^{e}S_{df}\right) & \beta \left(S_{k}-S_{df}\right)\\ 0 & 0 \end{array}\right]\left[\begin{array}{c} E_{k}\\ I_{k} \end{array}\right]\\ & =\left[\begin{array}{cc} 1-\mu & 0\\ \mu & 1-\gamma \end{array}\right]\left[\begin{array}{c} E_{k}\\ I_{k} \end{array}\right]+\left[\begin{array}{cc} \beta \lambda_{k}^{e}S_{k} & \beta S_{k}\\ 0 & 0 \end{array}\right]\left[\begin{array}{c} E_{k}\\ I_{k} \end{array}\right];\;k\in Z_{0+} \end{array}$$

Note that the spectral radius r of the matrix $\left[\begin{array}{cc} 1-\mu & 0\\ \mu & 1-\gamma \end{array}\right]$ is max(1−μ , 1−γ)<1 since μ∈[0, 1) and  γ∈[0, 1) and the above matrix is a convergent matrix. Since there is some matrix norm of arbitrary close value to the spectral radius there exist some positive real constants $\varepsilon \leq \delta_{0}-1+\min\left(\mu ,\gamma \right)$ and $r\leq \delta_{0}<1$ such that $\|\left[\begin{array}{cc} 1-\mu & 0\\ \mu & 1-\gamma \end{array}\right]\|\leq \delta_{0}$. In addition, since ${\left\{\beta_{k}S_{k}\right\}}_{k=0}^{\infty }\to \beta S_{df}=0$ and ${\left\{\lambda_{k}^{e}\right\}}_{k=0}^{\infty }\to \lambda^{e}$ and a decreasing nonnegative real sequence $\;{\left\{\delta_{1k}\right\}}_{k=0}^{\infty }\to 0$ having a strictly decreasing subsequence ${\left\{\delta_{1N_{n_{k}}}\right\}}_{k=0}^{\infty }\to 0$ such that for any given $\;\delta_{1N_{n_{k}}}$, there exists a non-negative integer $N_{n_{k}}$ such that $\left\|[\begin{array}{cc} \beta \lambda_{k}^{e}S_{k} & \beta S_{k}\\ 0 & 0 \end{array}]\right\|\leq \delta_{1N_{k}}$; $j=N_{n_{k}},N_{n_{k}+1},\ldots ,N_{n_{k+1}-1}$ such that ${\left\{N_{n_{k}}\right\}}_{k=0}^{\infty }$ is strictly increasing. Thus, one gets from (25) that:

(26) $$\|\left[\begin{array}{c} E_{N_{n_{k+1}}}\\ I_{N_{n_{k+1}}} \end{array}\right]\|\leq \delta_{0}^{N_{n_{k+1}}-N_{n_{k}}}\|\left[\begin{array}{c} E_{N_{n_{k}}}\\ I_{N_{n_{k}}} \end{array}\right]\|+\delta_{1N_{k}}\sum_{j=n_{k}}^{n_{k+1}-1}\delta_{0}^{n_{k+1}-1-j};\;k\in Z_{0+}$$

so that ${\left\{\left\|\left[\begin{array}{c} E_{N_{n_{k}}}\\ I_{N_{n_{k}}} \end{array}\right]\right\|\right\}}_{k=0}^{\infty }$ has a strictly decreasing subsequence which converges to zero for any given finite non-negative initial conditions. In view of (2)–(3), that property also implies that ${\left\{E_{k}\right\}}_{k=0}^{\infty }\to 0$ and ${\left\{I_{k}\right\}}_{k=0}^{\infty }\to 0$ for any given non-negative finite initial conditions and then (25) is globally asymptotically stable. Since it has been already proved that ${\left\{S_{k}\right\}}_{k=0}^{\infty }\to 0$ then $x_{df}={\left(0,\;0,0,R_{df}=N_{df}\right)}^{T}$ is globally asymptotically stable and ${\left\{N_{k}\right\}}_{k=0}^{\infty }\to N_{df}$. □

The next result gets condition for which the stationary model has a stable infective substate, irrespective of the susceptible being zero at the equilibrium point or not. It is found that there exists a critical transmission rate under which the stationary substate of the infective model is globally asymptotically stable. Under the extra conditions from Theorem 3, the critical transmission rate becomes infinity since the susceptible subpopulation is zero at the disease-free equilibrium point. As a result, the disease-free equilibrium point is globally asymptotically stable for any value of the transmission rate, and there is no endemic attractor.

**Theorem** **4.** *Assume* μ∈[0 , 1) *and* γ∈[0 , 1) *. Then, the stationary infective substate (25) has stable characteristic roots if* $\beta \in \left[0,\;\beta_{c}\;\right)$ *, where* $\beta_{c}=\frac{\mu \gamma }{{\left(\mu +\lambda^{e}\gamma \right)}^{}S_{df}}$ *so that it is globally asymptotically stable.*

*In addition, if the conditions of Theorem 2 hold with*$K\neq \frac{a}{\rho_{1}+\rho_{2}}$*then*$\beta_{c}=+\infty$.

*The stable solution sequence is guaranteed to be, furthermore, non-negative for any non-negative finite initial conditions if all the conditions of Theorem 3 hold.*

**Proof.**  If ${\left\{\beta_{k}S_{k}\right\}}_{k=0}^{\infty }\to \beta S_{df}=s$ and ${\left\{\lambda_{k}^{e}\right\}}_{k=0}^{\infty }\to \lambda^{e}$, then the characteristic Equation of (25) is:

(27) $$\lambda^{2}+b\lambda +c=0$$

where

(28) $$b=b\left(s\right)=\mu +\gamma -\left(2+\lambda^{e}s\right)$$

(29) $$c=c\left(s\right)=\left(1-\mu +\lambda^{e}s\right)\left(1-\gamma \right)-\mu s$$

Note that b(0)=μ+γ−2<0 and c(0)=(1−μ)(1−γ)>0. The coefficients of the polynomial defining the characteristic equation for s=0, ordered in decreasing order, have two changes of sign. According to Descartes’ rule of signs, such a polynomial has either two or zero positive real roots. Note that by simple inspection or, equivalently, according to the unforced part of (25), the characteristic Equation (27) has in fact two positive real characteristic roots for s=0 within the unit complex circle centered at zero which are $\lambda_{1}=1-\mu$ and $\lambda_{2}=1-\gamma$. Now, by inspection, $b\left(s\right)=b\left(0\right)-\lambda^{e}s<0$ for all s≥0. It is now discussed a valid range of non-negative values of s which guarantees that $c\left(s\right)=c\left(0\right)+\left(\lambda^{e}\left(1-\gamma \right)-\mu \right)s$ remains to be positive. Write by convenience $s=\frac{\sigma \mu \gamma }{\mu +\lambda^{e}\gamma }$. Then, c(s)>0 if and only if $\hat{c}\left(\sigma \right)>0$, where: $\hat{c}\left(\sigma \right)=\left(\mu +\lambda^{e}\gamma \right)c\left(s\right)=\left(\mu +\lambda^{e}\gamma \right)c\left(0\right)-\sigma \mu \gamma \left(\mu -\lambda^{e}\left(1-\gamma \right)\right)>0$ for any real σσ if $\lambda^{e}\geq \mu /\left(1-\gamma \right)$ with the eventual negative real values having no interest since s≥0 and, if $\lambda^{e}<\mu /\left(1-\gamma \right)$ then $\hat{c}\left(\sigma \right)>0$ if

$$\sigma <\frac{\mu +\lambda^{e}\gamma }{\mu -\lambda^{e}\left(1-\gamma \right)}\frac{\left(1-\mu \right)\left(1-\gamma \right)}{\mu \gamma }=\frac{\mu +\lambda^{e}\gamma }{\mu +\lambda^{e}\gamma -\lambda^{e}}\left(\frac{1-\mu -\gamma }{\mu \gamma }+1\right)=1+\sigma_{0}$$

for some real $\sigma_{0}>0$. In both cases, that is, irrespective of $\lambda^{e}$, c(s)>0 if $s=\frac{\sigma \mu \gamma }{\mu +\lambda^{e}\gamma }$ and σ∈[0 , 1]. Since there are two real positive characteristic roots for s=0 from Descartes rule of signs (since b(0)<0 and c(0)>0) and since the characteristic roots are continuous functions of the argument s and b(s)<0 and c(s)>0 for $s=\frac{\sigma \mu \gamma }{\mu +\lambda^{e}\gamma }$ with σ∈[0 , 1], one concludes that the characteristic roots are real (and positive) if $s=\beta S_{df}<\frac{\mu \gamma }{\mu +\lambda^{e}\gamma }$. It remains now to prove that those real positive roots are within the open unit circle. The two (real) within the complex open circle or radius unity centered at the origin of the complex plane if and only if both roots of (27), subject to (28)–(29), are within the open unit circle centered at zero in the complex plane, namely if and only if:

(30) $$-1<\lambda_{1,2}=\frac{2-\mu -\gamma +\lambda^{e}s\pm \sqrt{{\left(2-\mu -\gamma +\lambda^{e}s\right)}^{2}+4\left(\mu s-\left(1-\mu +\lambda^{e}s\right)\left(1-\gamma \right)\right)}}{2}\;<1$$

equivalently, if and only if,

(31) $$\begin{array}{cc} \mu +\gamma -\lambda^{e}s-4 & <-\sqrt{\lambda^{e^{2}}s^{2}+{\left(\gamma -\mu \right)}^{2}+4\mu s+2\lambda^{e}s\left(\gamma -\mu \right)}\\ & \leq \sqrt{\lambda^{e^{2}}s^{2}+{\left(\gamma -\mu \right)}^{2}+4\mu s+2\lambda^{e}s\left(\gamma -\mu \right)}<\mu +\gamma -\lambda^{e}s \end{array}$$

equivalently, if and only if,

(32) $$\sqrt{\lambda^{e^{2}}s^{2}+{\left(\gamma -\mu \right)}^{2}+4\mu s+2\lambda^{e}s\left(\gamma -\mu \right)}<\min\left(\mu +\gamma -\lambda^{e}s\;,\;4+\lambda^{e}s-\mu -\gamma \right)=\mu +\gamma -\lambda^{e}s$$

since the last equality follows directly since μ,γ∈(0 , 1). Taking the squares in both sides of (32) yields that it is equivalent to

(33) $$\lambda^{e^{2}}s^{2}+{\left(\gamma -\mu \right)}^{2}+4\mu s+2\lambda^{e}s\left(\gamma -\mu \right)<\lambda^{e^{2}}s^{2}+{\left(\mu +\gamma \right)}^{2}-2\lambda^{e}s\left(\mu +\gamma \right)$$

and also equivalent to

(34) $${\left(\gamma -\mu \right)}^{2}+4\mu s+2\lambda^{e}s\left(\gamma -\mu \right)<{\left(\mu +\gamma \right)}^{2}-2\lambda^{e}s\left(\mu +\gamma \right)$$

and, furthermore, equivalent to

(35) $${\left(\gamma -\mu \right)}^{2}+4\mu s+4\lambda^{e}s\gamma <{\left(\mu +\gamma \right)}^{2}$$

which holds if and only if

(36) $$s=\beta S_{df}<\frac{\mu \gamma }{\mu +\lambda^{e}\gamma }$$

Since $s=\beta S_{df}\geq 0$, the constraint (36) implies that the characteristic Equation (27) of the stationary infective substate (25) has stable roots if $\beta \in \left[0,\;\beta_{c}\;\right)$, where $\beta_{c}=\frac{\mu \gamma }{{\left(\mu +\lambda^{e}\gamma \right)}^{}S_{df}}$. Now, assume that all the conditions of Theorem 2 hold. Since $S_{df}=0$ if $K\neq \frac{a}{\rho_{1}+\rho_{2}}$ then $\beta_{c}=+\infty$ if $K\neq \frac{a}{\rho_{1}+\rho_{2}}$ and the stationary infective substate (25) has stable roots if β∈[0, +∞ ).

In addition, any solution is non-negative for all time if (15) and assumption 1 plus any of the four remaining assumptions 2 to 5 of Theorem 3 holds (according to Theorem 3). Thus, the joint non-negativity and global asymptotic stability of the disease-free stationary solution, under any finite non-negative initial conditions, are guaranteed for β∈[0, +∞ ) since $S_{df}=0$ (Theorem 3). □

Note that the Descartes rule of signs used in the proof of Theorem 4 is supported by the fact that for s=0 both characteristic roots are real, positive and stable, so that the possibility of having no positive root is excluded by such a value of the argument s, and the fact that the characteristic roots are continuous functions of s, so that they continue to be within the positive region of the unity circle centered at zero as the argument increases.

Note also that the proposed model is claimed for its usefulness for short-term predictions in the evolution phase when the disease is blowing up. It is neither considered that vaccination is available for use nor that there is immunity lost allowing to increase again the susceptible numbers after a certain delay. Therefore, the evolution of the susceptible subpopulation is given by a decreasing sequence. It is now proved that the proposed model does not have an endemic equilibrium point.

## 4. Nonexistence of Endemic Equilibrium Point

It is now discussed under which reasonable conditions an endemic equilibrium point $x_{end}={\left(S_{end},\;E_{end}\left(\neq 0\right),\;I_{end}\left(\neq 0\right)\;,\;R_{end}\right)}^{T}$ either exists or it does not exist. As a final result, it is concluded that the endemic equilibrium point cannot exist.

Consider the following possible cases:

**Case** **1**$\left(S_{end}>0\right)$. Combining (2) and (3) for stationary limiting model parameters for an assumed to exist endemic equilibrium point leads to provided that $E_{end}\neq 0$ and $S_{end}\neq 0$:

(37) $$I_{end}=\frac{\mu }{\gamma }E_{end}$$

(38) $$S_{end}=\frac{\gamma \mu }{\beta \left(\mu +\lambda^{e}\gamma \right)}>0$$

which, replaced in (1) and after cancelling common factors with $S_{end}>0$, results in the resulting constraint:

(39) $$S_{end}={\left[a-\beta \left(I_{end}+\lambda^{e}E_{end}\right)-K\left(\rho_{1}+\rho_{2}\right)\right]}^{}S_{end}$$

leads to

(40) $$E_{end}=\frac{a-1-K\left(\rho_{1}+\rho_{2}\right)}{\beta \left(\mu +\gamma \lambda^{e}\right)}\gamma$$

if

(41) $$a\neq \beta \left(I_{end}+\lambda^{e}E_{end}\right)+K\left(\rho_{1}+\rho_{2}\right)$$

so that $E_{end}>0$ (and also $I_{end}>0$ from (37)) if and only if $K<\frac{a-1}{\rho_{1}+\rho_{2}}\leq 1$ and $\;1<a\leq 1+\rho_{1}+\rho_{2}\leq 2$. If $K=\frac{a-1}{\rho_{1}+\rho_{2}}$ and $1\leq a\leq 1+\rho_{1}+\rho_{2}$ then $E_{end}=I_{end}=0$ so that the equilibrium point is, in fact, a disease-free one. By considering also the stationary recovered, one gets after cancelling the stationary recovered $R_{k+1}=R_{k}=R_{end}$ in both sides of (4) that the subsequent constraint should hold:

(42) $$\gamma I_{end}+K\left(\rho_{1}+\rho_{2}\right)S_{end}=\mu E_{end}+K\left(\rho_{1}+\rho_{2}\right)S_{end}=0$$

which contradicts that $S_{end}>0$ and $E_{end}>0$. As a result, no endemic equilibrium point exists with $S_{end}>0$. Since (42) can only hold with $E_{end}=S_{end}=0$ then, it only could eventually be true for the disease-free equilibrium point. Thus, Case 1 is unfeasible for the existence of an endemic equilibrium point.

**Case** **2**$\left(S_{end}\geq 0\right)$. Note that (40) holds with, which includes also $S_{end}>0$ of Case 1 if

(43) $$a=\beta \left(I_{end}+\lambda^{e}E_{end}\right)+K\left(\rho_{1}+\rho_{2}\right)=\beta \left(\frac{\mu }{\gamma }+\lambda^{e}\right)E_{end}+K\left(\rho_{1}+\rho_{2}\right)$$

provided that

(44) $$E_{end}=\frac{a-K\left(\rho_{1}+\rho_{2}\right)}{\beta \left(\mu +\gamma \lambda^{e}\right)}\gamma$$

provided that

(45) $$K<\frac{a}{\rho_{1}+\rho_{2}}\leq 1;\;a\leq \rho_{1}+\rho_{2}$$

with $S_{end}=0$, and

(46) $$I_{end}=\frac{\mu }{\gamma }E_{end}=\frac{a-K\left(\rho_{1}+\rho_{2}\right)}{\beta \left(\mu +\gamma \lambda^{e}\right)}\mu$$

However, again, after cancelling $R_{k+1}=R_{k}=R_{end}$ in both sides of (4), one gets that the subsequent constraint should hold:

(47) $$\gamma I_{end}+K\left(\rho_{1}+\rho_{2}\right)S_{end}=\mu E_{end}+K\left(\rho_{1}+\rho_{2}\right)S_{end}=0$$

which implies that $S_{end}=E_{end}=I_{end}=0$ (which only holds if $K=\frac{a}{\rho_{1}+\rho_{2}}$), and it is in fact a disease- free equilibrium point. In this case, one also has that $N_{end}=R_{end}$. Thus, Case 2 is unfeasible for the existence of an endemic equilibrium point.

**Remark** **3** (basic reproduction number)**.** *In biological terms, it is possible to re-interpret the condition of asymptotic stability around the disease-free equilibrium point in terms of the basic reproduction number, which indicates the number of secondary infectious individuals generated from one primary infectious one, defined by*

(48) $$R=\frac{\beta }{\beta_{c}}=\frac{\beta \left(\mu +\lambda^{e}\gamma \right)S_{df}}{\mu \gamma }$$

*It turns out that*R<1*is exactly identical to*$\beta <\beta_{c}$*, that is, if the transmission rate is smaller than its critical value*$\beta_{c}$*then, equivalently, the basic reproduction number is smaller than unity. That asymptotic stability condition, in the two mentioned equivalent forms, are related to the asymptotic extinction of the disease. It can be commented that such a condition is not related to the transient disease evolution but to its steady-state which is the disease-free equilibrium in this case. We can also to define a sample-dependent effective reproduction number as follows:*

(49) $$R_{k}=\frac{\beta \left(\mu +\lambda_{k}^{e}\gamma \right)S_{k}}{\mu \gamma };\;k\in Z_{0+}$$

*whose meaning and evolution should not be confused with that of the basic reproduction number. This number can be greater than one at the beginning of the disease transmission (even if*R<1*) but*$R_{k}\to R$*as*k→∞*. Note also from (1) that*$R_{k}$*decreases faster (since*$S_{k}$*decreases faster) if vaccination is programmed than in the vaccination-free case. The effective reproduction number is usually periodically checked to elucidate the particular intervention measures to be taken depending on the disease transmission evolution. It can be also pointed out that*R=1*is related, in common situations associated with epidemic models, to the confluence of the disease-free equilibrium point with the endemic one while, for*R<1*, the disease-free equilibrium point is unstable and the endemic equilibrium point is an attractor for the solutions. According to the former discussion in this subsection, this model, which is proposed for short-term predictions, does not evaluate the influence possible endemic steady states of the disease so that the central discussion is related to the case when*R<1.

## 5. Simulation Results

**Figure 1:**
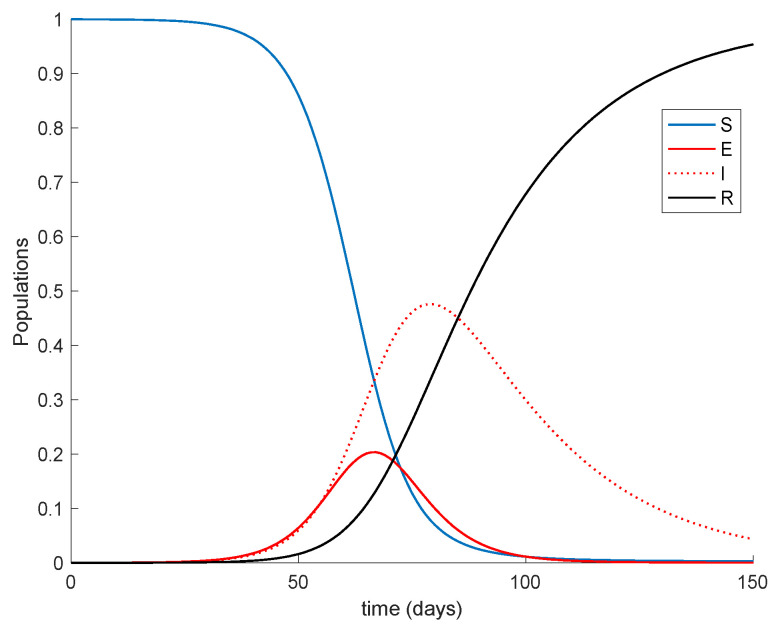
Dynamics of the model for COVID-19 pandemic.

This section contains some simulation examples devoted to study the application of the two-doses vaccination to counteract the spread of the COVID-19 pandemic. To this end the case of Italy borrowed from [45], where the discrete-model dynamics is confronted with actual data from Italy, will be considered. Thus, we have a discrete-time COVID-19 model serving as benchmark for the vaccination control. The model is parameterized by:

$$\beta =0.2,\beta^{e}=\frac{\beta }{1.3},\mu =\frac{1}{6},\gamma =0.04$$

so that $\lambda^{e}=0.7692$. The sampling time is one day so that the units of the parameters are in days^−1^. The initial conditions are $S_{0}=0.9999,E_{0}=0.0001,I_{0}=0,R_{0}=0$ implying that the total population is normalized to unity, without loss of generality. Notice that almost all the population is susceptible and a small fraction of the population is exposed at the beginning. The parameters of the vaccination are $d_{1}+d_{2}=21$ days and $d_{2}=7$ days; therefore, separation between the two doses is of two weeks. The values of the doses effectiveness are given by $\rho_{1}=0.66$ and $\rho_{2}=0.3$ in such a way that the total effectiveness of the two doses $\rho =\rho_{1}+\rho_{2}$ is 96%, in accordance with the average effectiveness of available vaccines. The natural recruitment rate is *a* = 1, since the natural growth of the population may be rejected when it comes to the epidemic spreading description due to the small number of children affected. The Figure 1 displays the dynamics of the model without vaccination.

**Figure 2:**
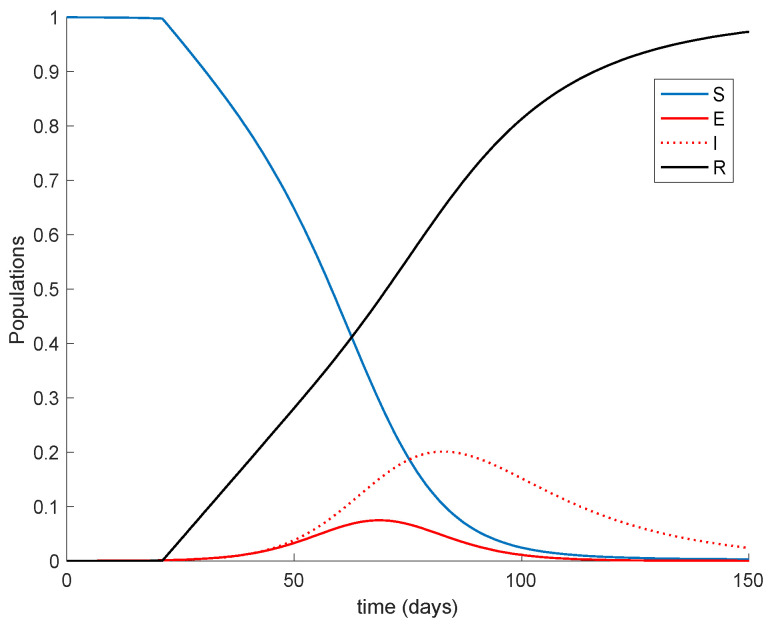
Dynamics of the model when two-doses vaccination is applied from the beginning.

It is remarkable in Figure 1 how the number of infectious reaches a peak at around the 50% of the total population. Now vaccination is applied with a gain of *K* = 0.01, corresponding to the 1% of the susceptible vaccinated every day. The vaccination is applied starting at different moments of the epidemic spreading in order to observe the effect of vaccination in the epidemic dynamics. Thus, Figure 2 displays the effect of vaccination when it is applied since the beginning of the spreading.

**Figure 3:**
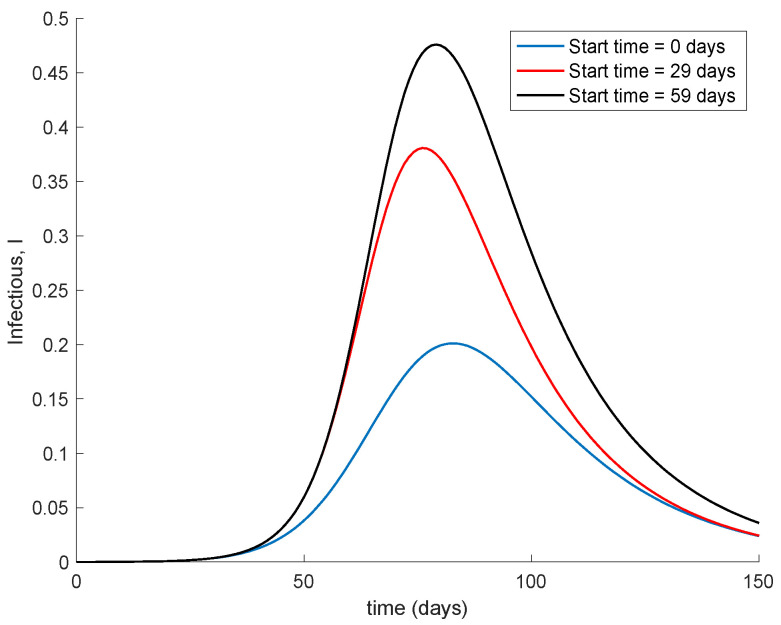
Effect of vaccination starting time on the infectious.

**Figure 4:**
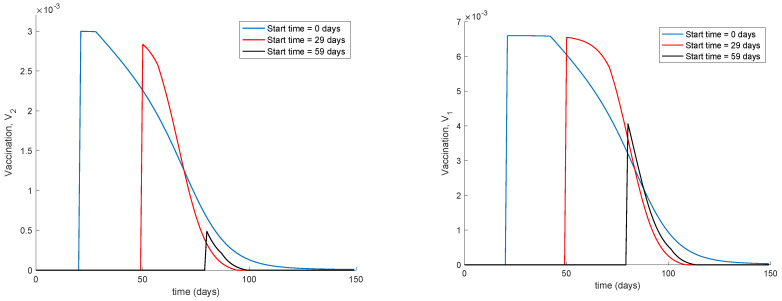
Vaccination actions corresponding to different starting times.

It is observed from Figure 1 and Figure 2 how the application of vaccination reduces the peak of the infectious while increasing the pace at which susceptible become immune, as expected. In addition, the Figure 3 illustrates the effect of starting the vaccination at different moments of the spreading ranging from the beginning, 29 days and 59 days after the first day of simulation. It can be seen in Figure 3 how the effect of vaccination in spreading is higher the sooner vaccination is applied when cases are detected. Thus, vaccination is useful for preventing new outbreaks and reducing their severity. If vaccination is applied when there is a relatively large number of cases among the population, the peak is not reduced ostensibly. Figure 4 displays the values of vaccination actions $V_{1}$ and $V_{2}$ corresponding to this situation. It is deduced from Figure 4 that the feedback vaccination provides a lower number of vaccinated individuals when it starts at advanced stages of the spread due to a smaller number of susceptible individuals. Therefore, this kind of action is especially recommendable when the spread is at its initial steps. Figure 4 also shows that vaccination $V_{1}$ has larger values than $V_{2}$ due to the differences in effectiveness defined by the parameters $\rho_{1}$ and $\rho_{2}$.

**Figure 5:**
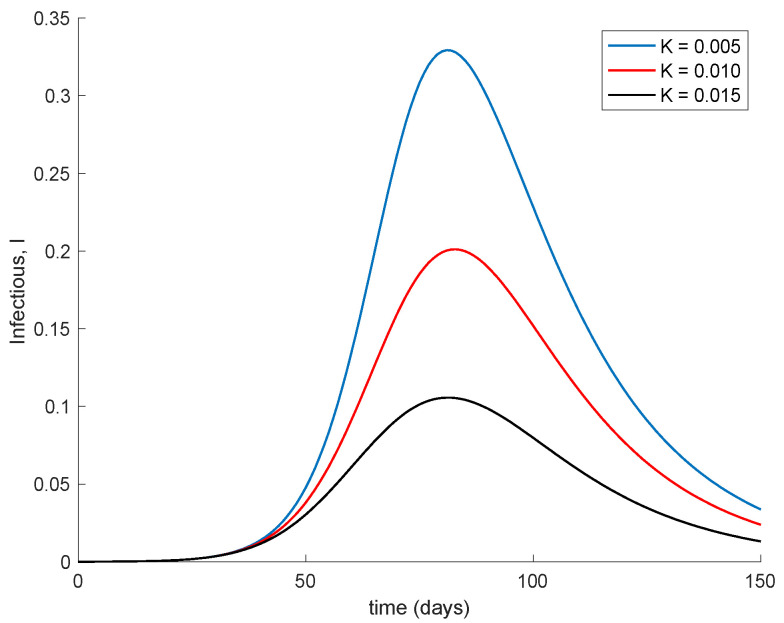
Dynamics of the infectious for different values of the vaccination gain.

**Figure 6:**
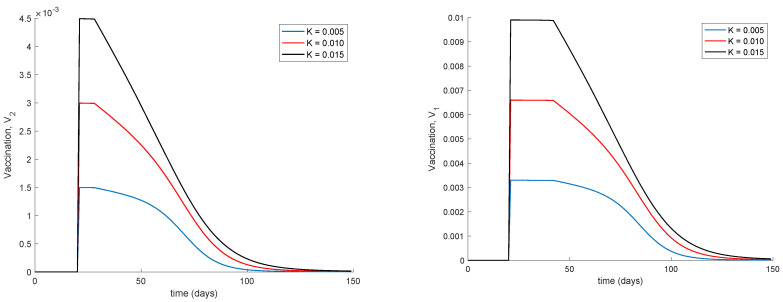
Vaccination actions for different values of the control gain *K*.

Finally, Figure 5 displays the dynamics of the model for different values of the vaccination gain. As it could have been expected, the larger the vaccination action is, the smaller peak is attained. Naturally, the reduction in the peak is achieved at the expense of a higher vaccination effort as Figure 6 shows. It can also be observed in Figure 4 and Figure 6 the effect of delay in the control action, where a value of zero vaccination is provided during the first days of simulation due to the delay.

**Figure 7:**
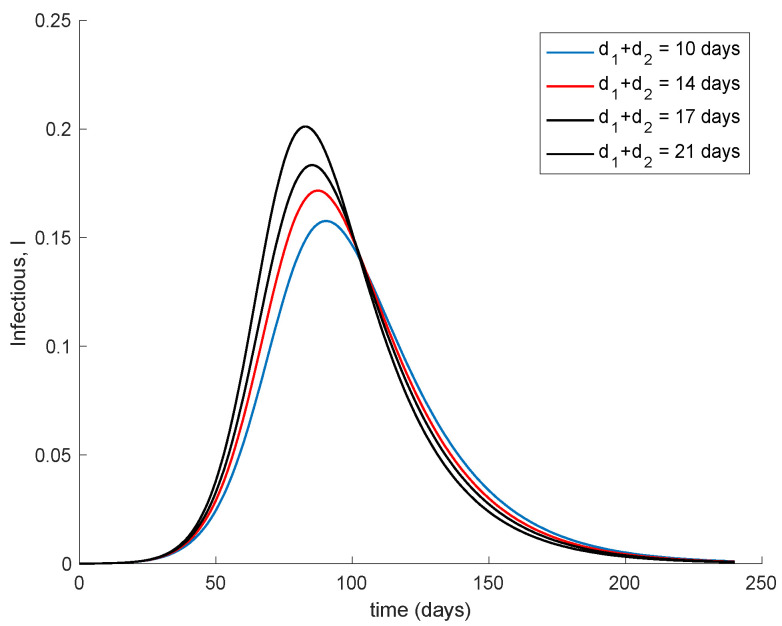
Dynamics of the infectious for different time periods between the two doses.

Figure 7 shows the effect of changing the interval of time between the application of the two doses. It is assumed that the effectivity does not change with the period in between them. The vaccination gain *K = 0.01* is used while the initial conditions are those indicated at the beginning of this section. Moreover, *d_2_ = 7* days and *d_1_ + d_2_* range from 10 to 21 days. It is observed in Figure 7 that if the second dose maintains effectivity regardless the dose sparing, it is better to administer it as soon as possible. However, in the real world, the effectivity of the second dose may depend on when this is applied. There are no data on how the effectivity depends on the dose sparing or these are scarce, [46]. Therefore a more accurate simulation on the effect of dose sparing is not carried out in this work.

**Figure 8:**
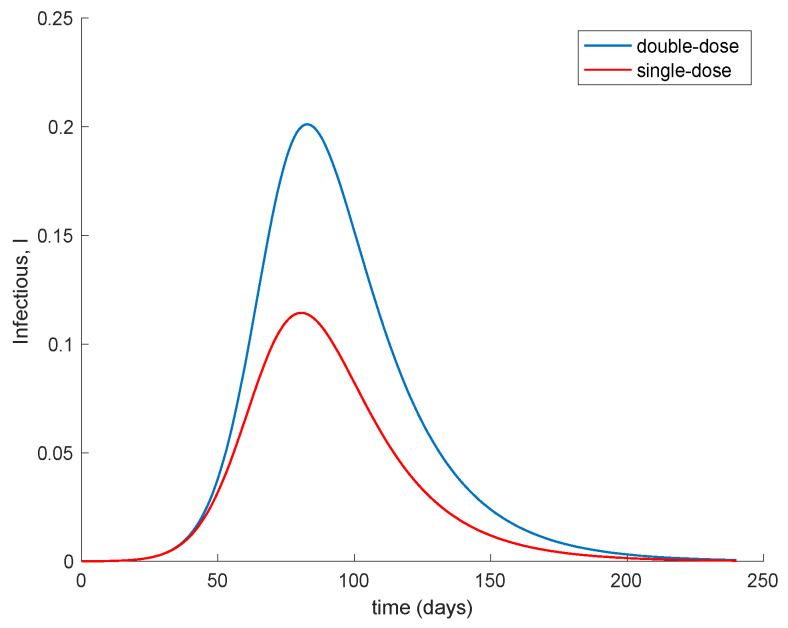
Comparison in the evolution of the number of infectious between the administration of a double dose (*K* = 0.01) or a single dose to a broader population (*K* = 0.02).

Moreover, Figure 8 compares the evolution of the infectious when two doses with a spacing of 14 days is applied (*K* = 0.1) in contrast of applying a single dose with a double-vaccination gain (*K* = 2 × 0.01 = 0.02). The Figure 8 shows that with respect to the evolution of the infectious the administration of a single dose to a broader population alleviates the peak of infectious in comparison to promptly applying the second dose in order to increase the overall effectiveness of the vaccine. This behavior is behind the decision of the UK government of prioritizing the first dose to as many people as possible while delaying the second dose, [46].

**Figure 9:**
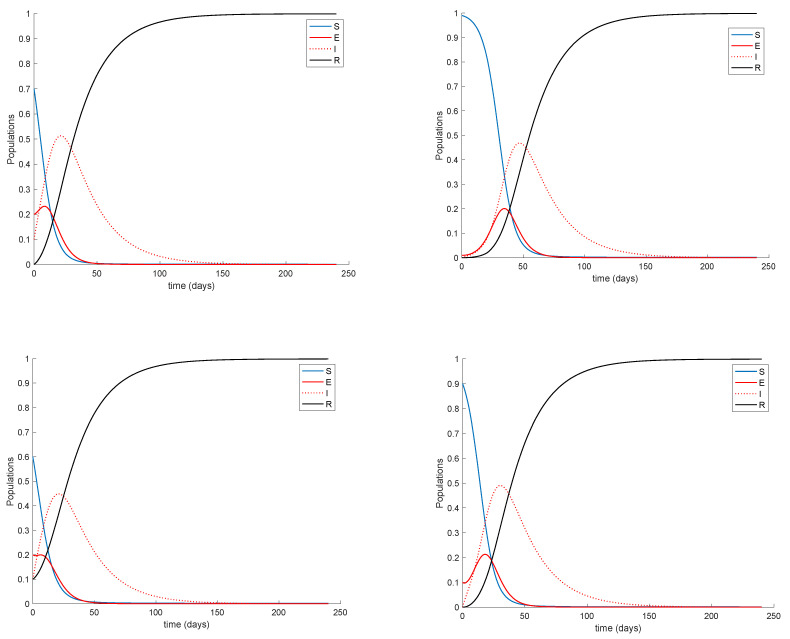
Dynamics of the model for different initial conditions and *K* = 0.001.

**Figure 10:**
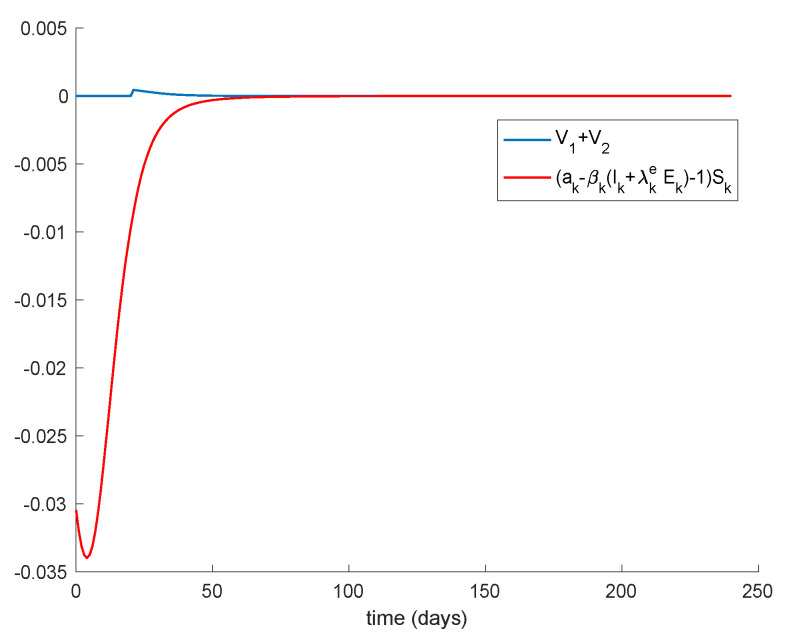
Satisfaction of Condition 3 from Theorem 3.

The basic properties of the model discussed in Section 3 and Section 4 can also be corroborated in the previous examples. Thus, the number of exposed and infectious always converges to zero so that the model converges to the disease-free equilibrium point. This happens because there is no endemic point for this model as discussed in Section 4. The Figure 9 displays a longer simulation for the model with *K* = 0.001 and different initial conditions. It is shown in Figure 9 that the model always converges to the globally-stable disease-free equilibrium point given by $S_{df}=E_{df}=I_{df}=0,R_{df}=1$ and all the subpopulations remain non-negative at all time. These properties are indeed guaranteed by Theorems 1 and 2 since μ,γ∈[0,1) and $\beta =0.2<1.66=\max\left(\frac{a_{k}}{I_{k}+\lambda_{k}^{e}E_{k}}\right)$ is small enough; therefore, (15) holds, and the model trajectory solution is non-negative according to Theorem 1 and converges to the disease-free equilibrium point according to Theorem 2 since $K=0.001\neq 1.04=\frac{a}{\rho_{1}+\rho_{2}}$. In addition, Theorem 3 also ensures that the disease-free equilibrium point is globally stable since Condition 1 from Theorem 3 is satisfied, Condition 2 also holds as $K=0.001\neq 1.04=\frac{a}{\rho_{1}+\rho_{2}}$ and Equation (13) holds as Figure 10 depicts. Therefore, the disease-free equilibrium point is concluded to be globally asymptotically stable.

It can be pointed out that different alternative theoretic methods for stability analysis of dynamic systems in the presence of delays can be found, for instance, in [47,48,49] and some of the references therein.

## 6. Concluding Remarks

A new discrete SEIR model has been presented in this paper subject to two delayed doses of feedback vaccination controls on the susceptible. It is also assumed that there exists a transmission rate from the exposed to the susceptible and that the transmission rates exposed-susceptible and infectious-susceptible may eventually be distinct. The second idea is based on the knowledge that the later period in the asymptomatic incubation phase of the infection in the COVID-19 pandemic is also infective as it is the first phase of the infectious period. The first idea of using two vaccination doses is based on the administration mechanisms of some of the recently approved vaccines for COVID-19. Such two doses are applied with a certain interval, and the immunity effect is delayed in respect to each current picked- up sample on the model, while it is also assumed that both doses can have, in general, different effectiveness. It is assumed, in particular, that the second dose has an incremental benefit on the injection of the first single dose. The effectiveness degrees of both doses as well as the delays can be adjusted in the experimental tests since they are model parameters. The non-negativity properties of the solution under finite non-negative initial conditions, its boundedness and the disease-free equilibrium point as well as its stability properties are also investigated. The non-negativity of the solution can become lost for large-enough transmission rates, but it is kept for low and moderate values of the transmission rates. The proposed model has been numerically tested through numerical examples for COVID-19 parameterizations.

## Data Availability

All data used are in the references and properly cited within the manuscript.

## Appendix A

### Appendix A1. Some Auxiliary Technical Results

**Theorem** **A1.** *Consider the difference equation:*

(A1) $$x_{k+1}=ax_{k}-K\left(\rho_{2}x_{k-d_{2}}+\rho_{1}x_{k-d_{1}-d_{2}}\right)+u_{k};\;k\in Z_{0+}$$

*with*$\rho_{1},\rho_{2}\in R_{+}$, a∈R,K∈R, *nonnegative(integer) delays*$d_{1}>0$*and*$d_{1}+d_{2}>d_{1}$*, and initial conditions*$x_{-d_{1}-d_{2}}=x_{-d_{2}}=0$*and*$x_{0}\in R$*and eventually subject to a bounded forcing sequence*${\left\{u_{k}\right\}}_{k=0}^{\infty }\subset R$*. Then, the following properties hold:*

(i) *The unforced difference Equation (A1), i.e., if*${\left\{u_{k}\right\}}_{k=0}^{\infty }\equiv 0$*, is globally asymptotically stable, so that*${\left\{x_{k}\right\}}_{k=0}^{\infty }$*is bounded and*${\left\{x_{k}\right\}}_{k=0}^{\infty }\to 0$*for any given finite*$x_{0}\in R$*, if*$K\in {\left(\max\left(\frac{a-1}{\rho_{1}+\rho_{2}}\;,\;-\overset{—}{K}\right)\;,\;\min\left(\frac{1+a}{\rho_{1}+\rho_{2}}\;,\;\overset{—}{K}\right)\right)}^{}$*and, in particular,*$K\in \left[0\;,\;\overset{—}{K}\right)$*under the restriction*$K\in R_{0+}=R_{+}\cup \left\{0\right\}$*, where:*
  $$\overset{—}{K}=\sup\{y\in \left(\frac{a-1}{\rho_{1}+\rho_{2}}\;,\;\frac{1+a}{\rho_{1}+\rho_{2}}\right)\;:y<1/f\left(y\right)\}\;with\;f:R_{0+}\to R_{0+}\;defined\;by:$$
  $$f\left(y\right)=\underset{\theta \in \left(0,2\pi \right)}{sup}\sqrt{\frac{{\left(\rho_{2}\left(\cos\left(\theta d_{2}\right)-1\right)+\rho_{1}\left(\cos\left(\theta \left(d_{1}+d_{2}\right)\right)-1\right)\right)}^{2}+{\left(\rho_{2}\sin\left(\theta d_{2}\right)+\rho_{1}\sin\left(\theta \left(d_{1}+d_{2}\right)\right)\right)}^{2}}{1+{\left(a-\left(\rho_{1}+\rho_{2}\right)y\right)}^{2}-2\left(a-\left(\rho_{1}+\rho_{2}\right)y\right)\cos\theta }}$$
(ii) *The unforced difference Equation (A1) is globally asymptotically stable for any given finite*$x_{0}\in R$*, so that*${\left\{x_{k}\right\}}_{k=0}^{\infty }$*is bounded and*${\left\{x_{k}\right\}}_{k=0}^{\infty }\to 0$*for any finite*$x_{0}\in R$*, if*|a|<1*and*$K\in \left(-\overset{—}{K}\;,\;\overset{—}{K}\right)$*, were*$\overset{—}{K}=1/\underset{\theta \in \left[0,2\pi \right)}{sup}\left|\frac{\rho_{2}e^{-i\theta d_{2}}+\rho_{1}e^{-i\theta \left(d_{1}+d_{2}\right)}}{e^{i\theta }-a}\right|$.
(iii) *Assume that either the conditions of Property (i) or those of Property (ii) hold and that*${\left\{u_{k}\right\}}_{k=0}^{\infty }\to 0$*. Then,*${\left\{x_{k}\right\}}_{k=0}^{\infty }$*is bounded and*${\left\{x_{k}\right\}}_{k=0}^{\infty }\to 0$*for any given finite initial condition*$x_{0}$.
(iv) *Assume that either the conditions of Property (i) or those of Property (ii) hold and that*${\left\{u_{k}\right\}}_{k=0}^{\infty }\;$*is bounded. Then,*${\left\{x_{k}\right\}}_{k=0}^{\infty }$*is bounded for any given finite initial conditions.*

**Proof.** By using the one-step-ahead and the one-step-backward operators q and $q^{-1}$ defined, respectively, by $qx_{k}=x_{k+1}$ and $q^{-1}x_{k+1}=x_{k}$, one can compactly rewrite (A1) as follows by defining $K_{1}=K\rho_{1}$ and $K_{2}=K\rho_{2}$:

(A2) $$\left(q-a+K_{2}q^{-d_{2}}+K_{1}q^{-\left(d_{1}+d_{2}\right)}\right)x_{k}=u_{k}$$

The characteristic equation of the unforced system (A1) is derived from the left-hand-side of (A2) by taking into account the formal analogy between the z-transform operator and the one-step-ahead one and also multiplying the result by the factor $z^{d_{1}+d_{2}}$ to avoid the concourse of mixed contributions of the forward and backward operators. As a result, the characteristic equation of (A2) becomes:

(A3) $$\begin{array}{ll} p^{\prime }\left(z\right) & =z^{d_{1}+d_{2}}\left(z-a+K_{2}z^{-d_{2}}+K_{1}z^{-\left(d_{1}+d_{2}\right)}\right)\\ & =z^{d_{1}+d_{2}}\left(z-a+K_{1}+K_{2}+K_{2}\left(z^{-d_{2}}-1\right)+K_{1}\left(z^{-\left(d_{1}+d_{2}\right)}-1\right)\right)=0 \end{array}$$

The polynomial $p^{\prime }\left(z\right)$ is stable if its zeros are within |z|<1 and the circle is centered at the origin of unity radius. In view of (A.3), since $z^{d_{1}+d_{2}}$ has a zero of multiplicity $d_{1}+d_{2}$ in the center z=0 of the open circle |z|<1, the stability condition holds if

(A4) $$p\left(z\right)=z-a+K_{1}+K_{2}+K_{2}\left(z^{-d_{2}}-1\right)+K_{1}\left(z^{-\left(d_{1}+d_{2}\right)}-1\right)$$

has all its zeros in the open complex circle |z|<1. Note that $\rho_{i}=K_{i}/K(i=1,2)$ so that one gets from (A4) that:

(A5) $$p\left(z\right)=p_{0}\left(z\right)+\tilde{p}\left(z\right)$$

(A6) $$p_{0}\left(z\right)=z-a+\left(\rho_{1}+\rho_{2}\right)K\;;\;\tilde{p}\left(z\right)=K\left(\rho_{2}\left(z^{-d_{2}}-1\right)+\rho_{1}\left(z^{-\left(d_{1}+d_{2}\right)}-1\right)\right)$$

is stable if $-1<a-\left(\rho_{1}+\rho_{2}\right)K<1$ and if, in addition, $\left|\tilde{p}\left(z\right)\right|<\left|p_{0}\left(z\right)\right|$ for any z∈C on the circumference |z|=1, i.e., on the boundary of the circle |z|≤1: This follows from the Rouché′s theorem of zeros [40,41,42,43], which establishes that if all the zeros of $p_{0}\left(z\right)$ lie in |z|<1 (i.e., they are stable) and $\left|\tilde{p}\left(z\right)\right|<\left|p_{0}\left(z\right)\right|$ on |z|=1, which is trivially a Jordan curve, then all the zeros of $q\left(z\right)=p_{0}\left(z\right)+\tilde{p}\left(z\right)$ are also in |z|<1 since q(z) has the same number of zeros in |z|<1 as $p_{0}\left(z\right)$. The condition $\left|\tilde{p}\left(z\right)\right|<\left|p_{0}\left(z\right)\right|$ on |z|=1 is identical to the following implicit constraint:

(A7) $${\left|K\right|}^{-1}>\underset{\theta \in \left[0,2\pi \right)}{sup}\left|\frac{\rho_{2}\left(e^{-i\theta d_{2}}-1\right)+\rho_{1}\left(e^{-i\theta \left(d_{1}+d_{2}\right)}-1\right)}{e^{i\theta }-a+\left(\rho_{1}+\rho_{2}\right)K}\right|$$

after writing any complex number z with |z|=1 as $z=e^{i\theta }$ for some θ∈[0, 2π). By inspecting (A7), we note that, for θ=0, $\tilde{p}\left(0\right)=0$ so that θ=0 may be removed from the test (A7) so that by using cos(−θ)=cosθ and sin(−θ)=−sinθ; ∀θ∈[0,2π), we get:

(A8) $$1>\left|K\right|\underset{\theta \in \left(0,2\pi \right)}{sup}\sqrt{\frac{{\left(\rho_{2}\left(\cos\left(\theta d_{2}\right)-1\right)+\rho_{1}\left(\cos\left(\theta \left(d_{1}+d_{2}\right)\right)-1\right)\right)}^{2}+{\left(\rho_{2}\sin\left(\theta d_{2}\right)+\rho_{1}\sin\left(\theta \left(d_{1}+d_{2}\right)\right)\right)}^{2}}{{\left(\cos\theta -a+\left(\rho_{1}+\rho_{2}\right)K\right)}^{2}+\sin^{2}\theta }}$$

Then, Property (i) follows from (A8) combined with $-1<a-\left(\rho_{1}+\rho_{2}\right)K<1$, which jointly ensure that the unforced difference Equation (A1) has the property that ${\left\{x_{k}\right\}}_{k=0}^{\infty }\to 0$ for any finite $x_{0}$ by defining $f:R_{0+}\to R_{0+}$ by

(A9) $$f\left(y\right)=\underset{\theta \in \left(0,2\pi \right)}{sup}\sqrt{\frac{{\left(\rho_{2}\left(\cos\left(\theta d_{2}\right)-1\right)+\rho_{1}\left(\cos\left(\theta \left(d_{1}+d_{2}\right)\right)-1\right)\right)}^{2}+{\left(\rho_{2}\sin\left(\theta d_{2}\right)+\rho_{1}\sin\left(\theta \left(d_{1}+d_{2}\right)\right)\right)}^{2}}{1+{\left(a-\left(\rho_{1}+\rho_{2}\right)y\right)}^{2}-2\left(a-\left(\rho_{1}+\rho_{2}\right)y\right)\cos\theta }}$$

and by noting that $\sup\left\{y\in R_{0+}\;:y<\left(1/f\left(y\right)\right)\right\}\neq \emptyset$. The vaccination gain constraint is simplified to $K\in {\left[0\;,\;\overset{—}{K}\right)}^{}$ in the particular case that $K\in R_{0+}$ since the general constraint $K\in {\left(\max\left(\frac{a-1}{\rho_{1}+\rho_{2}}\;,\;-\overset{—}{K}\right)\;,\;\min\left(\frac{1+a}{\rho_{1}+\rho_{2}}\;,\;\overset{—}{K}\right)\right)}^{}$ implies that $-\overset{—}{K}<\frac{1-a}{\rho_{1}+\rho_{2}}$, that is, $\overset{—}{K}>\frac{a-1}{\rho_{1}+\rho_{2}}$. Since, furthermore, $\overset{—}{K}<\frac{a+1}{\rho_{1}+\rho_{2}}$, it follows that $\min\left\{\frac{a+1}{\rho_{1}+\rho_{2}}\;,\;\overset{—}{K}\right\}=\overset{—}{K}$ and if $K\in R_{0+}$ then the vaccination gain constraint is simplified to $K\in {\left[0\;,\;\overset{—}{K}\right)}^{}$. Moreover,$\;{\left\{x_{k}\right\}}_{k=0}^{\infty }$ is bounded since any convergent sequence is bounded. Since ${\left\{x_{k}\right\}}_{k=0}^{\infty }$ is bounded and ${\left\{x_{k}\right\}}_{k=0}^{\infty }\to 0$ for any finite $\;x_{0}$ then the unforced difference Equation (A1) is globally asymptotically stable. To prove Property (ii), rewrite (A5) as $p\left(z\right)=p_{01}\left(z\right)+{\tilde{p}}_{1}\left(z\right)$ where

(A10) $$p_{01}\left(z\right)=z-a;\;{\tilde{p}}_{1}\left(z\right)=K\left(\rho_{2}z^{-d_{2}}+\rho_{1}z^{-\left(d_{1}+d_{2}\right)}\right)$$

By using the same arguments that those in the proof of Property (i), it is found that the zeros of p(z) are in |z|<1 if −1<a<1 and

(A11) $${\left|K\right|}^{-1}>\underset{\theta \in \left(0,2\pi \right)}{sup}\left|\frac{\rho_{2}e^{-i\theta d_{2}}+\rho_{1}e^{-i\theta \left(d_{1}+d_{2}\right)}}{e^{i\theta }-a}\right|$$

after the replacement of (A7) by (A11). As a result, the unforced difference Equation (A1) is globally asymptotically stable. To prove, Property (iii), we first obtain from (A1) the following extended dynamic system of dimension $d_{1}+d_{2}+1$:

(A12) $${\hat{x}}_{k+1}=\hat{A}{\hat{x}}_{k}+{\hat{u}}_{k}$$

subject to initial conditions $x_{-i}=0$ for $i=1,2,\ldots ,d_{1}+d_{2}$, where

(A13) $${\hat{x}}_{k}={\left(x_{k},\;x_{k-1},\ldots ,x_{k-d_{2}},\ldots ,x_{k-d_{1}-d_{2}}\right)}^{T};\;{\hat{u}}_{k}={\left(u_{k},\;0,\ldots ,0,\ldots ,0\right)}^{T}$$

(A14) $$\hat{A}=\left[\begin{array}{cccc} a & 0\ldots 0 & -K\rho_{2} & \begin{array}{cc} 0\ldots 0 & -K\rho_{1} \end{array}\\ 1 & 0 & 0 & \begin{array}{cc} 0 & 0 \end{array}\\ \vdots & \vdots & \ddots & \begin{array}{cc} \vdots & \vdots \end{array}\\ 0 & 0 & 0 & \begin{array}{cc} 1 & 0 \end{array} \end{array}\right]$$

First note that all the eigenvalues of $\hat{A}$ are stable under the conditions of either Property (i) or Property (ii) since if ${\left\{x_{k}\right\}}_{k=0}^{\infty }\to 0$ if ${\left\{u_{k}\right\}}_{k=0}^{\infty }\equiv 0$ then ${\left\{{\hat{x}}_{k}\right\}}_{k=0}^{\infty }\to 0$ if ${\left\{{\hat{u}}_{k}\right\}}_{k=0}^{\infty }\equiv 0$ so that the unforced discrete dynamic system (A12) is globally asymptotically stable. Now, from recursive calculation using (A12), one gets:

(A15) $${\hat{x}}_{k+N}={\hat{A}}^{N}{\hat{x}}_{k}+\sum_{i=k}^{k+N-1}{\hat{A}}^{k+N-i-1}{\hat{u}}_{i};\;k,N\in Z_{0+}=Z_{+}\cup \left\{0\right\}$$

If ${\left\{u_{k}\right\}}_{k=0}^{\infty }\to 0$ then, for any given $\varepsilon \in R_{+}$, there exists N=N(ε) such that $\|{\hat{u}}_{i}\|=\left|u_{i}\right|\leq \varepsilon$; $\forall i\left(\in Z_{0+}\right)\geq N$. Then, for some $\hat{\alpha }\in \left(0,1\right)$ such that the complex circle centered at z=0 of radius $\hat{\alpha }$ contains all the eigenvalues of $\;\hat{A}$, one gets:

(A16) $$\|{\hat{x}}_{k+N_{k}}\|\leq M{\hat{\alpha }}^{N_{k}}\|{\hat{x}}_{k}\|+\sum_{i=k}^{\infty }M{\hat{\alpha }}^{k+N_{k}-i-1}\|{\hat{u}}_{i}\|\leq M{\hat{\alpha }}^{N_{k}}\|{\hat{x}}_{k}\|+M\frac{\varepsilon_{k}}{1-\hat{\alpha }}$$

for some (norm-dependent) real constant M≥1, for any given $k\in Z_{0+}$ and any given strictly decreasing sequence ${\left\{\varepsilon_{k}\right\}}_{k=0}^{\infty }\subset R_{0+}$, with $\varepsilon_{0}=\varepsilon$, and some corresponding strictly increasing sequence of integers ${\left\{N_{k}\right\}}_{k=0}^{\infty }$ such that $N_{k}=N_{k}\left(\varepsilon_{k}\right)$. Since ${\left\{\varepsilon_{k}\right\}}_{k=0}^{\infty }\to 0$ and ${\hat{\alpha }}^{N_{k}}\to 0$ as k→∞ then there exists the limit $\underset{k\to \infty }{\lim}{\hat{x}}_{k+N_{k}}=0$, so that $\underset{k\to \infty }{\lim}x_{k+N_{k}}=0$ as well, if $x_{0}$ is finite, and then ${\left\{x_{k}\right\}}_{k=0}^{\infty }\to 0$ and, since this sequence is convergent, it is also bounded. Property (iii) has been proved. Property (iv) follows by noting that the first inequality of (A16) partially holds if ${\left\{u_{k}\right\}}_{k=0}^{\infty }$ is bounded in the weakened form:

(A17) $$\|{\hat{x}}_{k}\|\leq M{\hat{\alpha }}^{k}\|{\hat{x}}_{0}\|+\sum_{i=0}^{\infty }M{\hat{\alpha }}^{k-i-1}\|{\hat{u}}_{i}\|\leq M{\hat{\alpha }}^{k}\|{\hat{x}}_{0}\|+\frac{M\varepsilon }{1-\hat{\alpha }}\leq M\|{\hat{x}}_{0}\|+\frac{C}{1-\hat{\alpha }}<+\infty ;\;\forall k\in Z_{0+}$$

(A18) $$\underset{k\to \infty }{\lim\;sup}\|{\hat{x}}_{k}\|\leq \frac{M\varepsilon }{1-\hat{\alpha }}<+\infty$$

for any given $k\in Z_{0+}$ and some real constant $+\infty >C\geq \underset{i\geq 0}{sup}\|{\hat{u}}_{i}\|=\underset{i\geq 0}{sup}|{\hat{u}}_{i}|$. □

**Corollary** **A1.**
*Consider the difference equation:*

(A19) $$x_{k+1}=a_{k}x_{k}-K_{k}\left(\rho_{2}x_{k-d_{2}}+\rho_{1}x_{k-d_{1}-d_{2}}\right);\;k\in Z_{0+}$$

*subject to initial conditions* $x_{0}\in R$ *and* $x_{-i}=0$ *for* $i=1,2,\ldots ,d_{1}+d_{2}$ *and assume that the limiting difference equation:*

(A20) $$x_{k+1}=ax_{k}-K\left(\rho_{2}x_{k-d_{2}}+\rho_{1}x_{k-d_{1}-d_{2}}\right)$$

*resulting from (A19) as* ${\left\{a_{k}\right\}}_{k=0}^{\infty }\to a$ *and* ${\left\{K_{k}\right\}}_{k=0}^{\infty }\to K$ *satisfies the conditions of the unforced Equation of system (A1) of Theorem A1 (i) and Theorem A1 (ii). Assume also that*

(A21) $$\left|K_{k}-K\right|=o\left({\hat{x}}_{k}{}^{-1}\right);\;\left|a_{k}-a\right|=o\left({\hat{x}}_{k}{}^{-1}\right)$$

*Then,* ${\left\{x_{k}\right\}}_{k=0}^{\infty }$ *is bounded and* ${\left\{x_{k}\right\}}_{k=0}^{\infty }\to 0$ *for any given finite initial conditions so that the difference Equation (A19) is globally asymptotically stable.*

**Proof.** Equation (A19) is of the form of Equation (A1) with

(A22) $$u_{k}=\left(a_{k}-a\right)x_{k}-\left(K_{k}-K\right)\left(\rho_{2}x_{k-d_{2}}+\rho_{1}x_{k-d_{1}-d_{2}}\right)$$

and ${\left\{u_{k}\right\}}_{k=0}^{\infty }\to 0$ under (A21). Thus, the proof is direct from Theorem A1 (iii). □

**Corollary** **A2.**
*Consider the difference Equation (A19) with* $x_{0}\geq 0$ *and* ${\left\{a_{k}\right\}}_{k=0}^{\infty }\subset \left[0,\;1\right]$ *, define* $\alpha_{k}=1-a_{k}$ *and assume that* ${\left\{\alpha_{k}\right\}}_{k=0}^{\infty }\to 0$ *,* $\overset{\infty }{\underset{k=0}{\sum }}\alpha_{k}=+\infty$ *and* $K_{k}=K-\frac{\mu_{k}}{\rho_{2}x_{k-d_{2}}+\rho_{1}x_{k-d_{1}-d_{2}}}$ *;* $\forall k\in Z_{0+}$ *, with* ${\left\{\mu_{k}\right\}}_{k=0}^{\infty }\subset \left[\left(\rho_{2}x_{k-d_{2}}+\rho_{1}x_{k-d_{1}-d_{2}}\right)K\;,+\infty \right)$ *being subject to the subsequent constraints:*
$\overset{\infty }{\underset{k=0}{\sum }}\left(\mu_{k}-\left(\rho_{2}x_{k-d_{2}}+\rho_{1}x_{k-d_{1}-d_{2}}\right)K\right)<+\infty$*;*$\mu_{k}=0$*if*$x_{k-d_{2}}x_{k-d_{1}-d_{2}}=0$*, and*$\mu_{k}=o\left({\left(\rho_{2}x_{k-d_{2}}+\rho_{1}x_{k-d_{1}-d_{2}}\right)}^{-1}\right)$. *Then,*${\left\{x_{k}\right\}}_{k=0}^{\infty }$*is bounded and*${\left\{x_{k}\right\}}_{k=0}^{\infty }\to 0$*for any given finite nonnegative initial conditions so that the difference Equation (A19) is globally asymptotically stable.*

**Proof.** The difference Equation (A19) is written equivalently as:

(A23) $$x_{k+1}=\left(1-\alpha_{k}\right)x_{k}+\mu_{k}-K\left(\rho_{2}x_{k-d_{2}}+\rho_{1}x_{k-d_{1}-d_{2}}\right)$$

with $\alpha_{k}=1-a_{k}$; $\forall k\in Z_{0+}$, ${\left\{a_{k}\right\}}_{k=0}^{\infty }\to 1$ since ${\left\{\alpha_{k}\right\}}_{k=0}^{\infty }\to 0$ and $\alpha_{k}\geq 0$, $\mu_{k}\geq 0$; $\forall k\in Z_{0+}$,$\overset{\infty }{\underset{k=0}{\sum }}\alpha_{k}=+\infty$ and $\overset{\infty }{\underset{k=0}{\sum }}\mu_{k}<+\infty$ since $\mu_{k}=o\left({\left(\rho_{2}x_{k-d_{2}}+\rho_{1}x_{k-d_{1}-d_{2}}\right)}^{-1}\right)$. Thus, it follows from Venter theorem [44] that ${\left\{x_{k}\right\}}_{k=0}^{\infty }\to 0$, and the sequence boundedness follows as a consequence of its convergence. □

**Theorem** **A2.**
*Consider the difference equation:*

(A24) $$x_{k+1}=a_{k}x_{k}+b_{k}$$

*with* $x_{0}\geq 0$ *, where the following constraints hold:*

(A25) $$a_{k}=1+{\tilde{a}}_{k},\;b_{k}=b+{\tilde{b}}_{k};\;b>0;\;\forall k\in Z_{0+}$$

(A26) $${\left\{{\tilde{a}}_{j}\right\}}_{j=0}^{\infty }\subset \left[0,\;\tilde{a}\right]\subset \left[0,\;+\infty \right),\;{\left\{{\tilde{b}}_{j}\right\}}_{j=0}^{\infty }\subset \left[0,\;+\infty \right)$$

(A27) $${\tilde{a}}_{k+1}<\frac{{\tilde{a}}_{k}}{a_{k}+b_{k}/x_{k}};\;\forall k\in Z_{0+},\;\sum_{k=0}^{\infty }{\tilde{b}}_{k}<+\infty$$

*Thus, the following properties hold:*

(i) *If*$x_{0}\neq 0$*then*${\left\{x_{k}\right\}}_{k=0}^{\infty }$*is unbounded. If*b=0*and the remaining above conditions hold, then*${\left\{x_{k}\right\}}_{k=0}^{\infty }$ is bounded.
(ii) *If*$x_{0}\neq 0$*,*${\left\{{\tilde{a}}_{j}\right\}}_{j=0}^{\infty }\subset \left[-1,\;0\right]$*,*$\left|{\tilde{a}}_{k+1}\right|>\frac{\left|{\tilde{a}}_{k}\right|}{a_{k}+b_{k}/x_{k}}$*,*b>0*,*${\left\{{\tilde{b}}_{j}\right\}}_{j=0}^{\infty }\subset \left[0,\;+\infty \right)$*and*$\overset{\infty }{\underset{k=0}{\sum }}{\tilde{b}}_{k}<+\infty$*then a necessary condition for*${\left\{x_{k}\right\}}_{k=0}^{\infty }\to 0$*is*$\overset{k}{\underset{j=0}{\sum }}{\tilde{a}}_{j}=-\infty$. *The result still holds if*${\left\{x_{k}\right\}}_{k=0}^{\infty }\to x\left(>0\right)$.

**Proof.** One gets recursively from (A24) and (A25) that

(A28) $$x_{k+1}-x_{0}-\left(k+1\right)b=\sum_{j=0}^{k}\left({\tilde{a}}_{j}x_{j}+{\tilde{b}}_{j}\right)$$

From the first inequality of (A27), (A24) and D′Alembert criterion for convergence of series on non-negative terms, one gets:

(A29) $${\tilde{a}}_{k+1}x_{k+1}<\frac{{\tilde{a}}_{k}x_{k}}{a_{k}x_{k}+b_{k}}x_{k+1}<\frac{{\tilde{a}}_{k}x_{k}}{x_{k+1}}x_{k+1}={\tilde{a}}_{k}x_{k}\Rightarrow \sum_{j=0}^{k}{\tilde{a}}_{j}x_{j}<+\infty ;\;\forall k\in Z_{0+}$$

so that ${\tilde{a}}_{k}x_{k}\to 0$ as k→∞. Now, (A29) together with the second condition of (A27) and (A28) leads to:

(A30) $$0\leq x_{k+1}-x_{0}-\left(k+1\right)b<+\infty ;\;\forall k\in Z_{0+}$$

the non-negative lower-bound in (A30) arising from the non-negativity of the right-hand-side of the equality (A28). The relations (A30) lead to

(A31) $$\underset{k\to \infty }{0\leq \lim\;sup}\left(x_{k+1}-x_{0}-\left(k+1\right)b\right)<+\infty$$

and also (A24) subject to (A26) and $x_{0}\geq 0$ implies that ${\left\{x_{k}\right\}}_{k=0}^{\infty }\subset clR_{0+}=R_{0+}\cup \left\{+\infty \right\}$. This concludes that $\underset{k\to \infty }{+\infty \leq \lim\;sup}x_{k}=+\infty$ from (A31) and b>0, which implies that there exists the limit $\underset{k\to \infty }{\lim\;}x_{k}=+\infty$ (otherwise, $\underset{k\to \infty }{\lim\;sup}x_{k}$ would be finite) and then the sequence ${\left\{x_{k}\right\}}_{k=0}^{\infty }$ is unbounded. If b=0 and the remaining constraints (A25)–(A27) hold then (A31) is modified to $\underset{k\to \infty }{0\leq \lim\;sup}\left(x_{k+1}-x_{0}\right)<+\infty$ implying that $\underset{k\to \infty }{x_{0}\leq \lim\;sup}\left(x_{k+1}\right)<+\infty$ which concludes that ${\left\{x_{k}\right\}}_{k=0}^{\infty }$ is bounded. Property (i) has been proved. To prove Property (ii), note that under the alternative given conditions, if ${\left\{x_{k}\right\}}_{k=0}^{\infty }\to 0$, then one gets from (A28), by taking into account that $\overset{\infty }{\underset{k=0}{\sum }}{\tilde{b}}_{k}=C_{b}<+\infty$ and D′Alembert’s criterion for divergence of series, that

(A32) $$-\infty =-\underset{k\to \infty }{lim}\left(x_{0}+kb\right)=\sum_{k=0}^{\infty }\left({\tilde{a}}_{k}x_{k}+{\tilde{b}}_{k}\right)\leq C_{b}+\sum_{k=0}^{\infty }{\tilde{a}}_{k}x_{k}\Rightarrow \sum_{k=0}^{\infty }\left|{\tilde{a}}_{k}\right|x_{k}=+\infty$$

and also $\left(\overset{\infty }{\underset{k=0}{\sum }}\left|{\tilde{a}}_{k}\right|\right)\underset{k\geq 0}{sup}x_{k}=+\infty$. Since ${\left\{x_{k}\right\}}_{k=0}^{\infty }$ is still non-negative and convergent, then it is bounded, and $0<\underset{k\geq 0}{sup}x_{k}<+\infty$ if $\overset{\infty }{\underset{k=0}{\sum }}\left|{\tilde{a}}_{k}\right|=+\infty$ and the result is easily seen to still hold if ${\left\{x_{k}\right\}}_{k=0}^{\infty }\to x\left(>0\right)$. The proof of Property (ii) is complete. □

### Appendix A2. A More Detailed Expansion of the Squared Numerator of (A9)

Note that Equation (A9) is of the form:

(A33) $$f\left(y\right)=\underset{\theta \in \left(0,2\pi \right)}{sup}g\left(\theta ,d_{1},d_{2},\rho_{1},\rho_{2},y\right)$$

for each given quadruple $\left(d_{1},d_{2},\rho_{1},\rho_{2}\right)$, where:

(A34) $$g\left(\theta ,d_{1},d_{2},\rho_{1},\rho_{2},y\right)=\sqrt{\frac{n\left(\theta ,d_{1},d_{2},\rho_{1},\rho_{2}\right)}{d\left(\theta ,\rho_{1},\rho_{2},y\right)}}$$

whose squared right-hand-side numerator $n\left(\theta ,d_{1},d_{2},\rho_{1},\rho_{2}\right)$ is expanded as follows:

(A35) $$\begin{array}{l} n\left(\theta ,d_{1},d_{2},\rho_{1},\rho_{2}\right)\\ \;\;={\left(\rho_{2}\left(\cos\left(\theta d_{2}\right)-1\right)+\rho_{1}\cos\left(\theta \left(d_{1}+d_{2}\right)\right)-1\right)}^{2}+{\left(\rho_{2}\sin\left(\theta d_{2}\right)+\rho_{1}\sin\left(\theta \left(d_{1}+d_{2}\right)\right)\right)}^{2}\\ \;\;=2\left(\rho_{2}^{2}+\rho_{1}^{2}\right)-2\rho_{2}^{2}\cos\left(\theta d_{2}\right)-2\rho_{1}^{2}\cos\left(\theta \left(d_{1}+d_{2}\right)\right)+2\rho_{1}\rho_{2}\left(1-\cos\left(\theta d_{2}\right)-\cos\left(\theta \left(d_{1}+d_{2}\right)\right)\right)\\ +2\rho_{1}\rho_{2}\left[\sin\left(\theta d_{2}\right)\left(\sin\left(\theta d_{2}\right)\cos\left(\theta d_{1}\right)+\cos\left(\theta d_{2}\right)\sin\left(\theta d_{1}\right)\right)+\cos\left(\theta d_{2}\right)\left(\cos\left(\theta d_{2}\right)\cos\left(\theta d_{1}\right)-\sin\left(\theta d_{2}\right)\sin\left(\theta d_{1}\right)\right)\right]\\ =2\left(\rho_{2}^{2}\left(1-\cos\left(\theta d_{2}\right)\right)+\rho_{1}^{2}\left(1-\cos\left(\theta \left(d_{1}+d_{2}\right)\right)\right)\right)+2\rho_{1}\rho_{2}\left(1+\cos\left(\theta d_{1}\right)-\cos\left(\theta d_{2}\right)-\cos\left(\theta \left(d_{1}+d_{2}\right)\right)\right) \end{array}$$

which leads to the following nested particular cases:

(a) If $d_{1}=d_{2}$ then, one gets by using $1-\cos\left(2\theta d_{2}\right)=2\sin^{2}\theta d_{2}$ that:
  $$n\left(\theta ,d_{1},d_{1},\rho_{1},\rho_{2}\right)=2\left(\rho_{2}^{2}\left(1-\cos\left(\theta d_{2}\right)\right)+2\rho_{1}\left(\rho_{1}+\rho_{2}\right)\sin^{2}\left(\theta d_{1}\right)\right)$$
(b) If $d_{1}=d_{2}$ and $\rho_{1}=\rho_{2}$ then
  $$n\left(\theta ,d_{1},d_{1},\rho_{1},\rho_{1}\right)=2\left(\rho_{1}^{2}\left(1-\cos\left(\theta d_{2}\right)\right)+4\rho_{1}^{2}\sin^{2}\left(\theta d_{2}\right)\right)$$
(c) If $d_{1}=d_{2}=0$ or $\rho_{1}=\rho_{2}=0$ then
  $$n\left(\theta ,0,0,\rho_{1},\rho_{1}\right)=n\left(\theta ,d_{1},d_{1},0,0\right)=0$$
(d) If θ=0 then $n\left(0,d_{1},d_{1},\rho_{1},\rho_{1}\right)=0$ which is not evaluated in the supremum over (0,2π) in (A.33). Note that the constraint y<1/f(y) to calculate $\overset{—}{K}$ in Theorem A1 always holds since 1/f(y)=+∞ for θ=0, 2π since $n\left(0,d_{1},d_{1},\rho_{1},\rho_{1}\right)=0$ so that it has not to be accounted for in the supremum evaluation as Theorem A1 formally establishes.

### Appendix A3. A Fast and Simple Delay-Dependent Stability Test Based on Rouché′s Theorem of Zeros Within Open Circles Contained in the Unit Circle

**Theorem** **A3.**
*The following properties hold:*

(i) *Let*a∈(0,1)*and consider a circle*|z|≤σ*of center z = 0 and of radius*σ∈(a , a+ε]*for some real*ε>0. *Assume that*$0<K<\frac{\left(\sigma -a\right)\sigma^{d_{1}+d_{2}}}{\rho_{1}+\rho_{2}\sigma^{d_{1}}}$. *Then, the unforced difference Equation (A1) has all its zeros in* a<|z|<σ. *As a result, if*a<1*and*0<ε≤1−a*then the unforced difference Equation (A1) has all its zeros in*a<|z|<1*so that it is stable.*
(ii) *Let*$c_{i}=c_{i}\left(d_{1},d_{2}\right)\in R_{+}$*for*i=1,2*, be chosen such that*$a^{d_{1}}<c_{1}\leq \leq \sigma^{d_{1}}\leq c_{2}$*(implying that*$\frac{\ln c_{1}}{\ln\sigma }-d_{2}<d_{1}\leq \frac{\ln c_{2}}{\ln\sigma }$. *Then, the unforced difference Equation (A1) has its zeros in*|z|<σ*if*
  $$0\leq K<\frac{\left(c_{1}^{-d_{1}}-a\right)c_{1}}{\rho_{1}+\rho_{2}c_{2}}.$$

**Proof.** One has from the first identity of (A3) that:

(A36) $$p^{\prime }\left(z\right)=p_{0}^{\prime }\left(z\right)+{\tilde{p}}^{\prime }\left(z\right)$$

where:

(A37) $$p_{0}^{\prime }\left(z\right)=z^{d_{1}+d_{2}}\left(z-a\right);\;{\tilde{p}}^{\prime }\left(z\right)=\left(\rho_{2}z^{d_{1}}+\rho_{1}\right)K$$

Consider a circle |z|≤σ of center z = 0 and of radius σ∈(a , a+ε] for some real ε>0. Any point of the circumference |z|=σ, i.e., the boundary of the circle |z|≤σ, is given by $z=\sigma e^{i\theta }$ for some θ∈[0 , 2π) with $i=\sqrt{-1}$ being the complex unity. It turns out that all the zeros of $p_{0}^{\prime }\left(z\right)$, which are z=a and z=0 (with multiplicity $d_{1}+d_{2}$), are in |z|<σ. From Rouché′s theorem, all the zeros of $p^{\prime }\left(z\right)$ are in a<|z|<σ if

(A38) $$\begin{array}{c} \sigma^{d_{1}+d_{2}}\sqrt{{\left(\sigma \cos\theta -a\right)}^{2}+\sigma^{2}\sin^{2}\theta }>K\sqrt{{\left(\rho_{2}\sigma^{d_{1}}\cos d_{1}\theta +\rho_{1}\right)}^{2}+\rho_{2}^{2}\sigma^{2d_{1}}\sin^{2}d_{1}\theta };\;\\ \theta \in \left[0,2\pi \right) \end{array}$$

or,

(A39) $$\sigma^{2\left(d_{1}+d_{2}\right)}\left(\sigma^{2}+a^{2}-2a\sigma \cos\theta \right)>K^{2}\left(\rho_{1}^{2}+\rho_{2}^{2}\sigma^{2d_{1}}+2\rho_{1}\rho_{2}\sigma^{d_{1}}\cos\left(d_{1}\theta \right)\right);\;\theta \in \left[0,2\pi \right)$$

The following cases can arise:

Case a) If $d_{1}$ is even then for θ∈[0,2π):
  (A40) $$\begin{array}{ll} \sigma^{2\left(d_{1}+d_{2}\right)}\left(\sigma^{2}+a^{2}\right) & >K^{2}\underset{\theta \in \left[0,2\pi \right)}{max}\left(\rho_{1}^{2}+\rho_{2}^{2}\sigma^{2d_{1}}+2\left(\rho_{1}\rho_{2}\sigma^{d_{1}}+a\sigma^{2\left(d_{1}+d_{2}\right)}\right)\cos\left(\theta \right)\right)\\ & =K^{2}\left(\rho_{1}^{2}+\rho_{2}^{2}\sigma^{2d_{1}}+2\left(\rho_{1}\rho_{2}\sigma^{d_{1}}+a\sigma^{2\left(d_{1}+d_{2}\right)}\right)\right)\\ & =K^{2}{\left(\rho_{1}+\rho_{2}\sigma^{d_{1}}\right)}^{2}+2a\sigma^{2\left(d_{1}+d_{2}\right)} \end{array}$$
  or, equivalently,
  $$K\left(\rho_{1}+\rho_{2}\sigma^{d_{1}}\right)<\sigma^{d_{1}+d_{2}}\left(\sigma -a\right)$$
Case b) If $d_{1}$ is odd then for θ∈[0,2π):
  (A41) $$\begin{array}{ll} \sigma^{2\left(d_{1}+d_{2}\right)}\left(\sigma^{2}+a^{2}\right) & >K^{2}\underset{\theta \in \left[0,2\pi \right)}{max}\left(\rho_{1}^{2}+\rho_{2}^{2}\sigma^{2d_{1}}+2\left(\rho_{1}\rho_{2}\sigma^{d_{1}}-a\sigma^{2\left(d_{1}+d_{2}\right)}\right)\cos\left(d_{1}\theta \right)\right)\\ & =K^{2}\left(\rho_{1}^{2}+\rho_{2}^{2}\sigma^{2d_{1}}+2\left|\rho_{1}\rho_{2}\sigma^{d_{1}}-a\sigma^{2\left(d_{1}+d_{2}\right)}\right|\right) \end{array}$$
  which is still guaranteed under the above condition which is equivalent to $0<K<\frac{\sigma^{d_{1}+d_{2}}\left(\sigma -a\right)}{\rho_{1}+\rho_{2}\sigma^{d_{1}}}$. The proof of Property (i) is complete. To prove Property (ii), note that if $c_{1}\leq \sigma^{d_{1}+d_{2}}\leq \sigma^{d_{1}}\leq c_{2}$ then $\left|a\right|<1/c_{1}^{d_{1}}<\sigma$. Those constraints guarantee that $0<K<\frac{\left(c_{2}^{-d_{1}}-a\right)c_{1}}{\rho_{1}+\rho_{2}c_{2}}$ implies that $0<K<\frac{\sigma^{d_{1}+d_{2}}\left(\sigma -a\right)}{\rho_{1}+\rho_{2}\sigma^{d_{1}}}$ which proves Property (ii) as a direct consequence of Property (i). □
